# Tracking Community Timing: Pattern and Determinants of Seasonality in *Culicoides* (Diptera: Ceratopogonidae) in Northern Florida

**DOI:** 10.3390/v12090931

**Published:** 2020-08-25

**Authors:** Agustin I. Quaglia, Erik M. Blosser, Bethany L. McGregor, Alfred E. Runkel, Kristin E. Sloyer, Dinesh Erram, Samantha M. Wisely, Nathan D. Burkett-Cadena

**Affiliations:** 1 Florida Medical Entomology Laboratory, University of Florida, 200 9th St. SE, Vero Beach, FL 32962, USA; erik.blosser@gmail.com (E.M.B.); alrunkel4@gmail.com (A.E.R.IV); ksloyer@ufl.edu (K.E.S.); derram@ufl.edu (D.E.); nburkettcadena@ufl.edu (N.D.B.-C.); 2 United States Department of Agriculture, 1515 College Ave., Manhattan, KS 66502, USA; Bethany.McGregor@usda.gov; 3 Department of Wildlife Ecology and Conservation, University of Florida, 110 Newins-Ziegler Hall, Gainesville, FL 32611, USA; wisely@ufl.edu

**Keywords:** *Culicoides*, EHDV, BTV, *Orbivirus*, phenology, vector free period, community ecology, vector-borne disease ecology, veterinary entomology, time series multivariate analysis

## Abstract

Community dynamics are embedded in hierarchical spatial–temporal scales that connect environmental drivers with species assembly processes. *Culicoides* species are hematophagous arthropod vectors of orbiviruses that impact wild and domestic ruminants. A better sense of *Culicoides* dynamics over time is important because sympatric species can lengthen the seasonality of virus transmission. We tested a putative departure from the four seasons calendar in the phenology of *Culicoides* and the vector subassemblage in the Florida panhandle. Two years of weekly abundance data, temporal scales, persistence and environmental thresholds were analyzed using a tripartite *Culicoides β*-diversity based modeling approach. *Culicoides* phenology followed a two-season regime and was explained by stream flow and temperature, but not rainfall. Species richness fit a nested pattern where the species recruitment was maximized during spring months. Midges were active year-round, and two suspected vectors species, *Culicoides venustus* and *Culicoides stellifer*, were able to sustain and connect the seasonal modules. Persistence suggests that *Orbivirus* maintenance does not rely on overwintering and that viruses are maintained year-round, with the seasonal dynamics resembling subtropical *Culicoides* communities with temporal-overlapping between multivoltine species. Viewing *Culicoides*-borne orbiviruses as a time-sensitive community-based issue, our results help to recommend when management operations should be delivered.

## 1. Introduction

Multihost disease systems are influenced by the community dynamics of hosts, vectors and the environment in which they live to facilitate parasite transmission that can vary over space and time [1,2,3,4,5,6,7]. In particular, spatio-temporal dynamics drive the phenological patterns of multihost parasite transmission, as defined by the space–time duality theory [8,9,10]. Influential temporal scales may include short-term processes such as flowering time, while species distribution patterns may be driven by long-term biogeographic processes such as the timing of the last glacial maxima. When modeling host–vector interactions that can be influenced by these varying spatio-temporal scales, we create a risk of mismatching these phenomena when scales are arbitrarily set [11]. Episystem analyses connect environmental parameters to the communities of hosts, vectors and parasites [12,13] and model transmission based on biotic interactions that sustain the system [14,15].

Vector phenology can drive the temporal patterns of disease emergence [4,16,17]. In general, the temporal distribution of the species-specific environmental thresholds influences demographic outcomes and the blood-feeding behavior of arthropod vectors, ultimately impacting transmission [18,19,20]. The transmission risk of *Borrelia burgdorferi* is affected depending on the time that the larval and nymphal *Ixodes scapularis* display host seeking activity, which is constrained by the timing in the larval and nymphal emergence. Sequential feeding, where nymphs first feed and transmit pathogens to hosts followed by larval feedings, results in greater Lyme transmission than when nymphs and larvae feed synchronously [21]. Further exploration of how vector phenology impacts other disease systems is warranted.

*Epizootic hemorrhagic disease* virus (EHDV) and *Bluetongue* virus (BTV) are *Orbivirus* in the family Reoviridae, globally distributed pathogens of ruminants that are transmitted by biting midges (Diptera: Ceratopogonidae: *Culicoides*) [22,23,24]. Collectively, these viruses cause hemorrhagic disease (HD) which is a key cause of mortality in North American cervid species [25,26] and critically impacts the white-tailed deer (*Odocoileus virginianus*) farming industry [27,28]. Multiple serotypes and strains of BTV and EHDV cocirculate in wild deer across much of the eastern and midwestern United States as well as sporadically in the western states [26,27,29]. *Culicoides* species are a diverse and ancient group of flies represented by ~1360 extant species [22]. The immature stages develop in wet soils and natural cavities filled by precipitation, while flying adults of both sexes exploit plant nectar sources for energy [30]. In addition, the adult females seek blood meals from vertebrates to complete their gonadotrophic cycle [24,30]. This trophic behavior coupled with seasonal population eruptions produces high attack rates on vertebrate hosts and facilitates the transmission of EHDV–BTV.

A complex suite of biotic and abiotic niche layers has been identified as driving *Culicoides* abundance where weather-related variables, landscape and host availability interact [31,32,33,34,35,36,37,38,39]. Other processes can also govern how communities are assembled. In the unified neutral theory of biodiversity, species are ecologically equivalent; meanwhile, differences in the dispersal ability and stochasticity in the demographic rate between species lead to processes such as speciation, extinction and immigration that shape diversity change [40]. However, it has not been studied if niche drivers in combination with neutral processes (hereafter, neutral drivers) can contribute to building up *Culicoides* seasonality [41,42,43,44]. Midge populations experience both seasonal variation and long-term temporal patterns. Therefore, transmission risk assessments should account for these fluctuations rather than assuming simple seasonal patterns [45,46,47,48,49,50].

The southeastern Coastal Plain of the US includes temperate climates in the panhandle, and subtropical–tropical climates along the Florida peninsula and the Gulf Coast creating a diversity of conditions for vector species [51]. Overall, 48 species of *Culicoides* occur in Florida [30]. The sole proven EHDV vector in US, *Culicoides sonorensis* Wirth and Jones, is uncommon within the state [52,53]. *Culicoides insignis* Lutz, is a demonstrated BTV vector [54] and suspected EHDV vector found throughout Florida and extending slightly into neighboring states [55,56]. In the panhandle, this species is too uncommon to account for much of the detected orbiviral disease. Field observations in the region suggest that multiple cooccurring species could be playing a vectorial role, and EHDV–BTV transmission could extend year-round. Across southeast US, large catches of *Culicoides debilipalpis* Lutz, *Culicoides stellifer* Coquillett, *Culicoides paraensis* Goeldi, *Culicoides haematopotus* Malloch, *Culicoides crepuscularis* Malloch and *Culicoides biguttatus* Coquillett have been found in accordance with HD outbreaks or near affected host species [29,57,58,59,60,61]. *Culicoides stellifer*, *C. venustus* and *C. debilipalpis* feed preferentially on competent hosts, but *C. haematopotus* feed heavily on birds and less often on ruminants [62,63]. In addition, several species have been found naturally infected at a low rate with BTV and EHDV (*C. crepuscularis*, *C. haematopotus, C. debilipalpis*, *C. stellifer, Culicoides furens* Poey, *Culicoides variipennis* Coquillet, *C. venustus)* [29,61,64,65]. Biological transmission of EHDV–BTV is feasible for *C. debilipalpis, C. venustus* and *C. stellifer*, although laboratory vector competence studies have been challenging to conduct, and results are inconclusive [57,59,66,67]. The great abundance of these species could overcome low overall infection rates that may be leading to the lack of successful virus isolations, especially if multiple competent vectors are present. Increasing our understanding of *Culicoides* community ecology in this region could guide our understanding of which species are likely to be contributing to pathogen transmission in a multivector environment.

In most temperate parts of the US, HD outbreaks are confined to August and September, but in Florida, virus detection can occur year-round [68,69,70]. In the region, HD cases are distributed between June and November, and virus circulation appears to be the highest from August to October when 90% of acute cases are detected [68,69]. The EHDV infection rate in *C. venustus* and *C. stellifer* has shown to be stable between August to October [61]. In addition, the detection of EDHV-positive animals and seroconversion between January and March in a sentinel herd of white-tailed deer shows that transmission could continue during winter months [70]. Because *Culicoides pallidicornis* Kieffer is a cold-tolerant species that feeds on competent hosts, [62] proposed that this species could lead to transmission outside the HD season. In addition, abundant species such as *C. insignis, C. stellifer* and *C. venustus* are multivoltine [71,72] and occur year-round, except for some winter weeks [45,46]. Accordingly, there is a knowledge gap for surveillance of *Culicoides* infection rates outside the HD season. The mechanisms that govern *Orbivirus* maintenance in this region have not been thoroughly investigated.

Here, we inspected how weekly to yearly *β*-diversity profiles of *Culicoides* communities in the panhandle of Florida intersect. The panhandle is unique in that it is where temperate and subtropical regimes have strong influences on local weather, and likely drive the year-round HD transmission [51]. The local *Culicoides* fauna encompasses Nearctic and Neotropical species [30] with different niche breath [38]. We hypothesize that *Culicoides* composition changes between dry and wet seasons but does not necessarily occur between the calendar seasons (spring, summer, fall and winter). Therefore, the *Culicoides* seasonality shows a mix of temperate and subtropical dynamisms and is reflective of regional weather patterns. Second, we hypothesize that spring months are a transition period for the coexistence of cold and warm tolerant species which facilitates virus transmission. Furthermore, mild winter temperatures contribute to the continuous persistence of EHDV-BTV in the episystem. These hypotheses were addressed with a free-scale tripartite community-based modeling approach. This analytical strategy not only allowed the discovery that the *Culicoides* community was conceived as the dynamic combination of co-occurring species in assemblages and environmental variables, it also provided information on the species level responses to the environment [11,57,58]. Our models elucidated the vector community assemblage and transmission drivers in this disease episystem. Results will assist in the timing of disease management operations aimed at the temporal dynamics in the *Culicoides* community [73,74].

## 2. Materials and Methods

### 2.1. Insect Collections

Biting midges were collected on a 180 ha game preserve located in Gadsden County, Florida. The preserve was located in the Gulf Coast Flatwoods ecoregion, the hardwood forest and the upland short pine (*Pinus taeda*) habitats dominated the landscape [27,75,76]. This preserve housed various ungulate species of the families Bovidae and Cervidae [62]. We trapped midges using ten CDC miniature UV light traps with 50 mL conical tubes containing 20 mL of 90% ethanol [61,62]. These methods have previously been shown to be suitable to quantify *Culicoides* diversity in Florida [77]. Ten traps were randomly stratified among habitats (Figure S1.1) using the ArcGIS v 10.3 (ESRI, Redlands, CA, USA), and operated twice weekly from July 2015 to June 2017 (101 weeks). In each habitat, four traps were used (hardwood forest: Traps # 2, 10, 11 and 16; upland short pine forest: Traps # 1, 12, 17 and 18) and two additional traps were used in the transition between both forest habitats (Traps # 5 and 15) (Figure S1.1). This design allowed us to explore the interaction between the temporal dynamics in the *Culicoides* community and the spatial distribution of the species in the preserve. The minimum distance between the traps was 328.7 m, and the minimum distance that connected all the traps was 605.6 m (Supplementary File 2; Figure S2.5). Between July and November, 10 more traps were placed in the property but not included in this study to ensure a balanced sampling effort between months. Timers in traps were set to begin collections one hour prior to sunset and turn off one hour after sunrise. The *Culicoides* collected were identified using morphological keys [30] and sorted by physiological status (nulliparous, parous, gravid or blood fed) following published methods [78,79].

### 2.2. Environmental Data

Daily temperature (average, maximum and minimum) and precipitation were obtained from the National Oceanic and Atmospheric Administration climate online datasets [80] (station USW00093805), and total stream discharge and average gage height were obtained from the United States Geographical Survey National Water Information System [81] (station USGS2330000). Lags were determined for 15, 30, 45 and 60 days prior to each trap date and included temperature, precipitation and stream data (environmental submodels).

### 2.3. Culicoides Data

Because day-to-day variability in *Culicoides* count is not relevant to the recruitment process, we used the day with the maximum trap catch each week in our analyses [47]. Data were consolidated into trap-week by a species abundance matrix (1010 observations: 101 weeks × 10 traps) where only the count of total identifiable females (i.e., irrespective of physiological stage) was used.

### 2.4. Culicoides β-Diversity Temporal Profile

We used a multivariate time series analysis of community composition to assess the assemblage of putative HD vectors across an annual cycle. A *β*-diversity analysis was implemented to test the departure of the *Culicoides* community from the four season temperate regimes (winter, spring, summer and fall), how environmental fluctuation constrained the assembling process and what other wider temporal scales (≥12 months) were observed. In addition, the distribution of the species among the space and habitat can be a confounder of the temporal change in community composition [9]. We did not observe independent temporal patterns in species composition among traps (Supplementary File 2). Summaries of species distribution among habitats were not reported here since the scope of this study was not to analyze the pattern of diversity partitioning among habitats.

**Figure 1:**
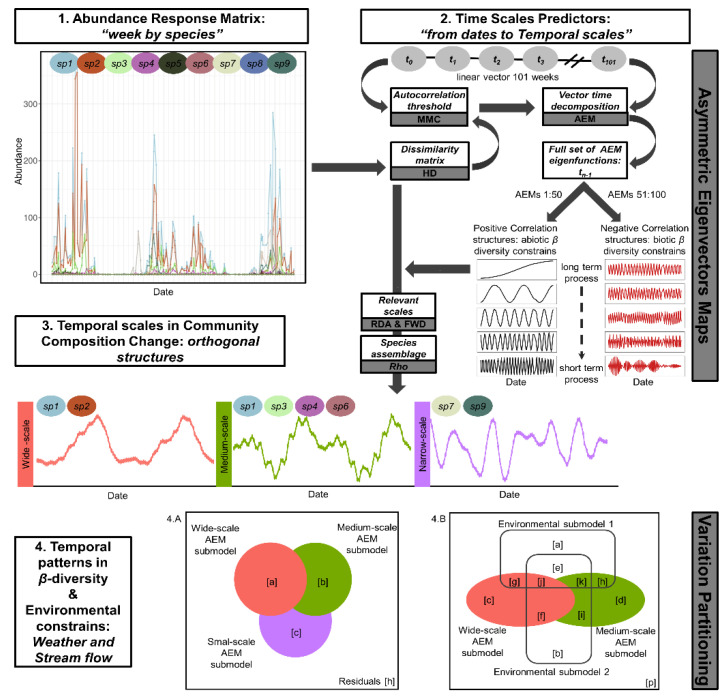
Workflow to analyze *Culicoides* temporal dynamic and its drivers: the sequence of steps from collecting *Culicoides* field abundance data, drawing temporal scales and identifying *β*-diversity scales and their relatedness with environmental variability. White filled boxes outline the aim for each analysis, and dark gray boxes mention the statistical tools used. Step 1 shows the temporal dynamic of the observed abundance in a nine-species virtual community; therefore, it was the response matrix informing weekly community composition. Step 2: the sampling temporal profile was thrown to spectral decomposition by asymmetric eigenvectors maps (AEMs) after accounting for the dependency between weeks (autocorrelation threshold). The resulting AEMs were half splitting in positive and negative correlation structures, and a subset was selected by their suitability to model temporal patterns in the observed abundance matrix. Selected subsets were arranged in groups representing broad to narrow temporal waves, hereafter referred to as temporal submodels. Step 3: the community composition change was modeled with each temporal submodel to assess the fitting of the temporal scale on the observed species abundance matrix. The assemblage of species contributing on each significant temporal scale was untangled by the correlation between each species abundance and Lc for each RDA_axis_. Step 4: the contribution of the temporal submodels and environmental variables to explain the amount of variation was visualized by Venn diagrams after hierarchical variation partitioning analysis. Since temporal scales were nested, even when there were orthogonal structures, both diagrams show intersection areas between temporal submodels as a hierarchical overlapping pattern. Letters inside diagrams are R^2^_adj_ for each unique and intersection fraction in the variation partitioning table (as were introduced in Section 2.4: *Culicoides β*-diversity temporal profile; equations for the fractions are detailed in [84]). AEM: asymmetric eigenvectors maps; AEMs: AEM eigenfunctions; FWD: forward selection; Hd: Hellinger’s distance; Lc: linear combinator scores from an RDA_axis_; MMC: multivariate Mantel correlogram; RDA: redundancy analysis; Rho: Rank Spearman correlation; R^2^_adj_: adjusted determination coefficient. This scheme was adapted considering [9,83] (Figure 1).

We employed four steps in the time series modeling framework (Figure 1). First, we averaged the trap-week abundance matrix (*n*>10) to a weekly abundance species matrix since spatial structures were not significant. This was the response matrix for the model (Figure 1, Step 1). Second, we derived self-informed temporal submodels from the species composition data and, as a regressor, the sampling temporal frame by asymmetric eigenvectors maps (AEM) (Figure 1, Step 2). Third, we assessed the fit of the temporal submodels operating on the observed species abundance matrix by redundancy analysis (RDA) to better understand how temporal scales influenced the models (Figure 1, Step 3). Fourth, we used hierarchical variation partitioning analysis (HVP) [82,83] to assess how the temporal scales and environmental variables explained species composition (Figure 1, Step 4).

A detailed explanation of the statistical toolbox and its expedience has been published elsewhere [9,10,11,85,86,87] and expanded on Supplementary File 2. Asymmetric eigenvectors maps were chosen because the temporal dependency is a forward directionally process that can be modeled by monotonic oscillation, and inferences about scales are independent [10,11,85]. The shortest independent time interval for community composition change was computed by multivariate Mantel correlogram (mMC) [87]. Positive (abiotic interactions) and negative (biotic interactions) eigenfunctions (AEMs) were selected separately by forward selection (FW) [88] and grouped representing wide (≥12 months), medium (≥6 months) and narrow temporal scales (1 week to 6 months). The fit of a scale’s submodel as a predictor of the Hellinger transformed [89] weekly abundance species matrix was tested by RDA. Variation within scales was judged by the retained profiles (RDA_axis_) after FW. With HVP, the relative importance of the environmental dimensions of the niche and neutral theory (birth, death and dispersal rates) drivers in *Culicoides β*-diversity was assessed as the unique fraction of variation from each environmental submodel (precipitation, temperature and stream level) and each temporal submodel, respectively. The additive fractions were used to judge how environmental constraints or demographic processes in the temporal scales could lead to the observed dynamics in *Culicoides* composition. The joint fractions explain the intercorrelation between scales and environment. The environmental submodels were selected by the FW using the HVP procedure [84] which allowed collinearity. The significance of the additive and unique fractions was tested by 1000 permutations, but there is no statistical procedure to test the joint fraction [87]. Negative values of R^2^_adj_ lack ecological meaning and were reported as R^2^_adj_ ≈ 0 [87]. Analyses were carried out in R 3.5.1 with vegan [90], adespatial [91] and adapted published codes [10,84,87].

### 2.5. Seasonality in Species Recruitment, Weekly Occurrence and Co-Occurrence Structure

We tested if spring months were a transition period for the coexistence of cold and warm tolerant *Culicoides* species by analyzing the relationship between diversity structure and species composition. Species recruitment across calendar seasons was analyzed using time to species saturation in *Culicoides* richness during spring with species–accumulation curves [92] in iNEXT [93]. As such, accumulation curves were fitted with rarefaction and extrapolation sampling curves for asymptotic Hill numbers (*q*_0_, *Chao_2_*) with the species by week incidence matrix. Additionally, we explored if the vector assemblage (*C. stellifer*, *C. venustus*, *C. debilipalpis*, *C. biguttatus* and *C. pallidicornis*) had sufficient sample coverage by season. With sample completeness curves [92], the number of weeks necessary to complete 97.5% of the vector species diversity was compared with the total sampled weeks during each season in iNEXT [93]. The linkage between richness and species composition was assessed to explore if the pattern of diversity change was related to loss or replacement of species using nestedness analysis [94] in vegan [90]. A nested pattern meant that species composition was structured within the season, where smaller communities form species subsets within larger communities [95]. Then, observations were sorted by maximizing the richness gradient through time and nestedness calculated using NODF (nestedness metric based on overlap and decreasing fill) [96]. Departure from randomness was tested as the deviance from a quasiswap generated null model (*n* = 1000).

The hypothesis that vectors are able to support year-round virus transmission was tested with a temporally explicit co-occurrence network. This analysis was selected in virtue of modeling simultaneously the probability of sharing time between *Culicoides* species at community level but also the case of EHDV–BTV vectors. Two species were considered to co-occur when they were detected the same week. First, those relevant occurrence interactions (OR > 3) in species pairwise association analysis constrained by temporal autocorrelation [97] were retained in an interaction matrix from the weekly species presence data (weeks: *n* > 0; species: *n* > 5, incidence > 5%) with sppairs [98]. Modularity (M) summarized the degree of compartmentalization in the species co-occurrence network [99] (i.e., seasons) and was defined by *Culicoides* species that co-occurred significantly more often with each other than with other species in the community and was estimated according to [100] in igraph [101]. In order to identify the role of each *Culicoides* species in bridging across seasonal modules, we identified four species role parameters that considered the persistence of a species in a seasonal module (within-module degree, *z*) and its ability to connect among them (among-module connectivity, *c*) [102] in bipartite [103]. The cut-off to define species role was fixed as [104]. *Culicoides* were defined as connectors (*z* < 1.3; *c* > 0.6) if they sustained network coherence by interacting among modules. *Culicoides* species that sustained the coherence of a module were considered a module hub (*z* > 1.3; *c* < 0.6). If a midge supported a module and increased network coherence, it was defined as a network hub (*z* > 1.3; *c* > 0.6). Peripheral *Culicoides* (*z* < 1.39; *c* < 0.6) co-occurred irregularly with species within a defined module and uncommonly interacted with other modules.

### 2.6. Environmental Thresholds in the Temporal Dynamics of the Vector Assemblage

Environmental thresholds in the temporal partitioning of putative HD vectors *C. stellifer, C. venustus, C. debilipalpis, C. biguttatus* and *C. pallidicornis* [61,62] were predicted by the monothetic clustering method multivariate regression tree (MVRT) [105] in mvpart [106]. This analysis allowed us to double-check if seasonality in the vector subassemblage and at community level (Section 2.4) overlapped. *Culicoides insignis* was not considered due to its scarcity on this property. Abundance data were Chord transformed [87,89] and regressed with the standardized temperature, precipitation and stream variables previously described. Clusters informed epidemiological times, and tree branch configuration was shaped by environmental thresholds. The output of the MVRT showed the sampled weeks organized in clusters that informed about epidemiological times as vectors abundance were regressed. Furthermore, the configuration of the tree relied on how the detected environmental thresholds explained the partitioning of the sampled weeks in clusters. To compute the environmental thresholds, the weeks frame was iteratively split into *n* clusters, and the value of the environmental variables that minimized the Euclidean distance within a cluster was selected. For the comparison of data between both years, weeks were standardized as epidemiological weeks. The first epidemiological week ended on the first Saturday of January, as long as it fell at least four days into the month. Cross validation (CV) of the tree was performed after 1000 iterations, and tree size was judged following [87]. Indicator species analysis (id) selected species which were statistically representative of each branch node [107].

## 3. Results

### 3.1. Insect Collections

Overall, 29 of 48 species of *Culicoides* known to occur in Florida were trapped among 52,136 individuals in 1010 traps/nights (Table S1.5) [30]. We did not observe any traps that had failed both nights in a week, and the weekly trap success (101 weeks) was sufficient to address the seasonal dynamics in *Culicoides* diversity (see also Section 3.3). The three more abundant species were *C. stellifer* (61.5%), *C. haematopotus* (13.5%) and *C. pallidicornis* (11.37%) (Table S1.5). Eight species represented less than 0.08% of the captured individuals and were removed from the analysis in Section 3.2 (*Culicoides bauri* Hoffman, *Culicoides beckae* Wirth and Blanton, *C. furens*, *Culicoides guttipennis* Coquillet, *Culicoides nanus* Root and Hoffman, *Culicoides ousairani* Khalaf, *Culicoides pusillus* Lutz, *Culicoides alachua/sanguisuga*). *Culicoides sonorensis* was not recorded in the ranch, and *C. insignis* was uncommon (0.13%). The density of vectors (midges/trap night) were skewed between weeks and species ($\bar{x}$ ± sd; *C. stellifer*: 31.8 ± 55.7; *C. pallidicornis*: 5.87 ± 20; *C. biguttatus*: 3.33 ± 9.85; *C. venustus*: 2.19 ± 2.47 and *C. debilipalpis*: 0.34 ± 0.84), and then, between and within seasonal variations were expected. Other potential vectors such as *C. crepuscularis* (0.04%), *C. paraensis* (0.07%) and *C. variipennis* (0.02%) were uncommon.

### 3.2. Culicoides β-Diversity Temporal Profile

**Figure 2:**
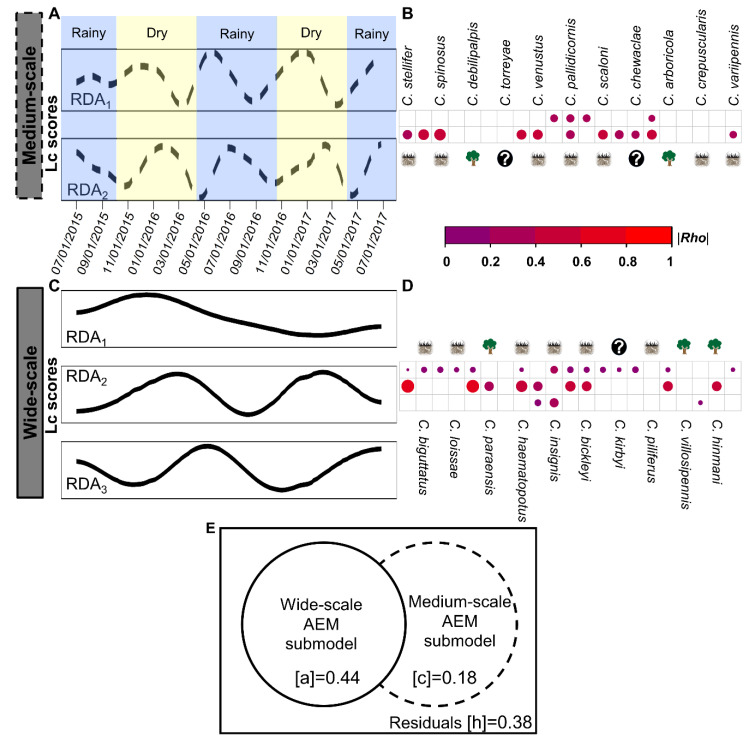
Temporal scales behind *Culicoides* assembling pattern: the changes in species composition followed annual and biannual temporal profiles. (**A**,**C**) Panels describe the weekly oscillation in the community composition by each significant AEM temporal submodel and the nonoverlapping structures within them. Table S1.1 summarizes temporal scales and their week range, AEMs and statistical significance. The x-axis is shared between panels (**A**,**C**). Panel A also shows the rainy (light blue) and dry (light yellow) seasons in Florida. (**B**,**D**) Panels indicate compromised species for the changing composition at each temporal profile (RDA) after Spearman rank correlation. Only significant |Rho| showed where size and color followed the magnitude of the species-temporal structure association (Table S1.2). Larval habitat traits are indicated as wet soils and tree holes with shapes. The question mark indicates those species where the larval habitat was not described. Panel (**E**): Venn diagram represents the amount of variation in the community change composition at each temporal scale by hierarchical variance partitioning. Fractions were described in *Culicoides β*-diversity temporal profile section and their R^2^_adj_ printed.

The *Culicoides* community showed changes in species composition that followed temporal patterns not necessarily defined by seasonality in temperate regions. The *Culicoides* community followed wide (4 AEMs; F = 20.33; df = 4; *p* = 0.001) and medium (7 AEMs; F = 3.32; df = 7; *p* = 0.001) temporal scales but did not fit a seasonal scale of less than 6 months (small-scale = 2 AEMs; F = 0.88; df = 2; *p* = 0.496). Medium-scale accounted for 18% of *Culicoides β*-diversity and produced two semiannual profiles (RDA_axis_; 24–29 weeks; 6–9 weeks lag; Figure 2A–E, Table S1.1) in accordance with the dry and wet seasons in subtropical Florida. Two assemblages of species drove dynamics at this scale (Figure 2B, Table S1.2). In the first assemblage, four species accounted for community change (|Rho| = 0.20–0.26). The second assemblage had the richest subassemblage (11 *Culicoides*; |Rho| = 0.24–0.58) and *C. biguttatus* and *Culicoides spinosus* Root and Hoffman showed the highest correlation. The wide-scale accounted for 44% of *Culicoides β*-diversity in two yearly profiles (10–19 weeks lag; Figure 2C–E, Table S1.1). *Culicoides stellifer*, *C. debilipalpis* and *C. haematopotus* Malloch played a dominant role (|Rho| = 0.56–0.72) in a 59-week profile, while *C. venustus, C. insignis* and *C.s crepuscularis* (|Rho| = 0.21–0.35) defined a 53 week profile (Figure 2D, Table S1.2). Another wide-scale profile (RDA_1_; 83 weeks; Figure 2C–E, Table S1.1) encompassed the 66% of the community composition (|Rho| < 0.25), but environmental data did not fit the model (Figure S1.3), and almost all the species were involved in this profile (Figure 2D). The compositional changes that occurred in the temporal windows of nine or fewer weeks were autocorrelated (Figure S1.2), and biotic interactions were not observed (negative AEMs; Table S1.1). In general, *Culicoides β*-diversity was composed of a series of phenological modules which described sequences of nonoverlapping temporal scales and species composition.

**Figure 3:**
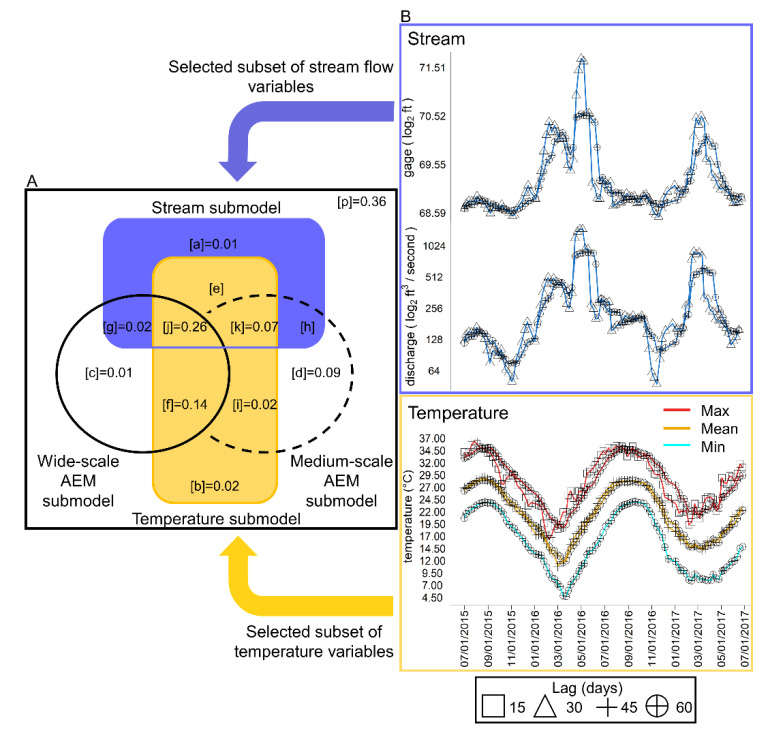
*Culicoides* dynamics are shaped by environmental and time-related constraints. (**A**) describes the proportion of weekly *Culicoides* composition variation concurrently explained by environmental (stream submodel and temperature submodel) and temporal (wide- and medium-scale submodels) determinants. The continuous line represents the wide-scale temporal submodel, and the discontinuous line is the medium-scale temporal submodel. The yellow color diagram represents the temperature submodel, and the stream derived submodel is light blue. Each fraction shows the corresponding R^2^_adj_ and [e] and [h] R^2^_adj_ *≈ 0*. Unique and additive fractions were significant after permutations analysis (*n* = 1000, *p <* 0.01). (**B**) Observed fluctuations in stream (upper blue panel) and temperature (yellow bottom panel) selected variables (metrics and days-lag) are plotted against sampling weeks. Then, the stream submodel was comprised of the mean gauge height (ft; upper side of stream metrics panel) and discharge flow (ft^3^/second; bottom side of stream metrics panel) at 30 and 60 days lag by the forward selection scheme proposed. Both are showed in *log_2_* scale for improving visualization. The temperature submodel comprises minimum (cyan line; 60 days lag), maximum (golden line, 15 and 30 days lag) and mean (red line, 45 and 60 days lag) metrics after forward selection.

Tracking variation in the community composition across time revealed that both environmental and temporal constraints emerged to mold the pattern of the *Culicoides* community, explaining 64% of the observed variation (Figure 3A). Half of the overall variation accounted for the intercorrelation between neutral and niche drivers (environmental and temporal submodels joint effect; [j]R^2^_adj_ = 0.26; [k]R^2^_adj_ = 0.07). Precipitation did not contribute in the environmental submodel (Figure 3B) as neither of the lags fit the species dissimilarity matrix after FW. The unique (stream: [a]R^2^_adj_ = 0.01, F = 2.21; df = 20; *p* < 0.01; temperature: [b]R^2^_adj_ = 0.02, F = 2.22; df = 20; *p* < 0.01) and additive (R^2^_adj_ = 0.54, F = 2.91; df = 20; *p* < 0.01) contribution of the stream and temperature submodels were significant. Notably, the contribution of neutral and niche drivers into *Culicoides β*-diversity was scale dependent and illustrated how temporal scales and the environment interacted. First, the environmental submodel dominated the wide-scale profile ([c]R^2^_adj_ = 0.01, F = 2.47; df = 20; *p* < 0.01) as 97% of the total variation arose from the joint effect of temperature and stream conditions ([g]R^2^_adj_ = 0.02; [f]R^2^_adj_ = 0.14; [j]R^2^_adj_ = 0.26). Second, neutral and environmental drivers were constrained equally at the medium-scale (50%; stream: [h]R^2^_adj_ ≈ 0; temperature: [i]R^2^_adj_ = 0.02; [k] R^2^_adj_ = 0.07). Moreover, neutral drivers were nine times higher in the medium-scale ([d]R^2^_adj_ = 0.09, F = 2.91; df = 20; *p* < 0.01) than wide-scale ([c]). Dynamics of the *Culicoides* composition across time were shaped by measurable niche conditions (stream flow fluctuations and atmospheric temperature, but not precipitation) and neutral drivers assembling over unique timeframes.

### 3.3. Seasonality in Species Recruitment, Weekly Occurrence and Co-Occurrence Structure

**Figure 4:**
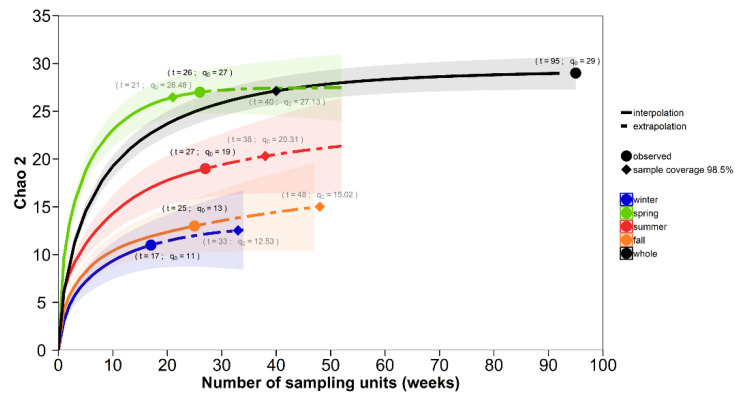
Seasonality pattern in *Culicoides* community structure: richness profile is trigged during spring. Sampled size-based rarefaction and extrapolation plot of *Culicoides* are shown to depict the seasonality richness pattern. Species accumulation curves (*Chao_2_*, *q0*) across the sampling weeks in each season (colors) are drawn. The solid line describes rarefaction or interpolated curve. The dashed line represents the accumulation profile up to double the reference sample size (extrapolation curve). The 95% confidence interval is the shaded area, and significance between profiles was the inference of their nonoverlapping. Observed *q0* and the reference or complete sample size (*t*) are indicated with round dots and values detailed with bold legends. Diamonds, dots and grey legends show *q0* and *t* necessary to attain 98.5% of the expected richness after coverage-based rarefaction and extrapolation analysis.

**Figure 5:**
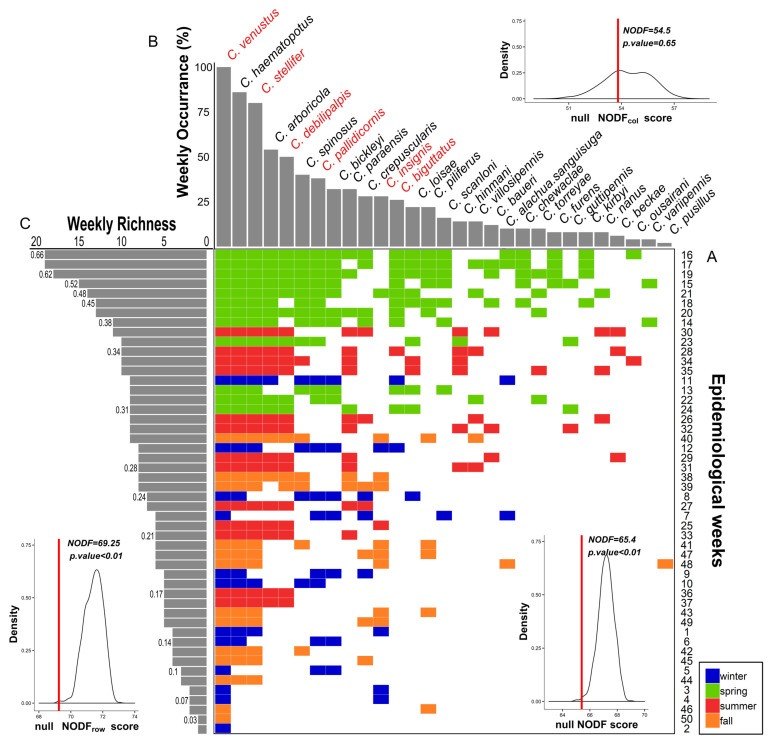
Composition and structure sharing schedule: species composition and *Culicoides* richness follow a nestedness pattern. (**A**) Pattern of nestedness is plotted in the central panel, where the assemblage at each epidemiological week is row-wise filled and the occurrence of species in column-wise on an incidence matrix. Rows and columns are arranged to maximize the decreasing fill and overlap (NODF) of the matrix. Cells color illustrates calendar season affiliation. Accordance between species occurrence ranking ((**B**), upper panel) and species arrangement in A is presented by a bar chart of observed species weekly occurrence. Red labeled species indicate *Culicoides* incriminated in *Orbivirus* transmission. Moreover, richness ((**C**), left panel) and weekly composition matching are illustrated by the weekly species richness bar chart parallelizing the week order in the nestedness matrix (**A**). Proportion of the total assemblage cover by each richness level is indicated at the left side of the bar. Inner density plot shows significance of NODF (red line) towards the null distribution of NODF scores (*n* = 1000).

Species recruitment (defined as the first appearance of a species) occurred during each seasonal compartment, and the highest recruitment occurred during the transition from the dry into the wet season (Figure 4 and Figure 5). Over the two-year study period, the species inventory was fully represented (*Chao_2_* = 29.49 (29.03; 37.37); sampling coverage (sc = 0.99)). Spring months were key to shaping *Culicoides* community structure, as interpolation–extrapolation curves show (Figure 4). Richness in spring nearly equaled richness of other seasons combined (*Chao_2_* = 27.48 (27.04–33.07), sc = 0.99) and richness achieved in any week in spring was greater (Figure 4) than any of the other seasons (Summer: *Chao_2_* = 22.85 (19.53–46.75); sc = 0.97; Fall: *Chao_2_* = 17.32 (13.47–52.37); sc = 0.97; Winter: *Chao_2_* = 13.12 (11.25–29.05); sc = 0.94). The rate of increase in the accumulated species across sampling weeks was the steepest during spring months (interpolation curve). During spring, richness was saturated before rarefaction ended. However, in summer months, the sampling effort had to increase 17%, 31% in fall and 32% in winter to reach 98.5% sampling coverage.

The sample completeness curve showed that in each season, 97.5% of the sampling coverage was reached as soon the field sampling effort reached 27 weeks (Figure S1.7). Consequently, the deployed sampling effort was enough to analyze the seasonal dynamics in the vector assemblage.

*Culicoides* richness and composition were linked through a pattern of nestedness, with the assemblage organized in a series of nested time-derived subassemblages (Figure 5A). Assemblages in weeks with low richness were formed with a limited set of species also occurring in high-richness weeks. Nestedness (NODF = 65.45; *p* < 0.01; matrix fill = 21%; Figure 5A) was more likely to be driven by richness and composition arrangement (NODF_row_ = 69.25; *p* < 0.01; Figure 5C) than the pattern of species occurrence (NODF_column_ = 53.80; *p* = 0.65; Figure 5B). Moreover, week order in the nested matrix depicted seasonality because species richness and composition followed a winter–fall–summer–spring gradient (subassemblage to full assemblage).

**Figure 6:**
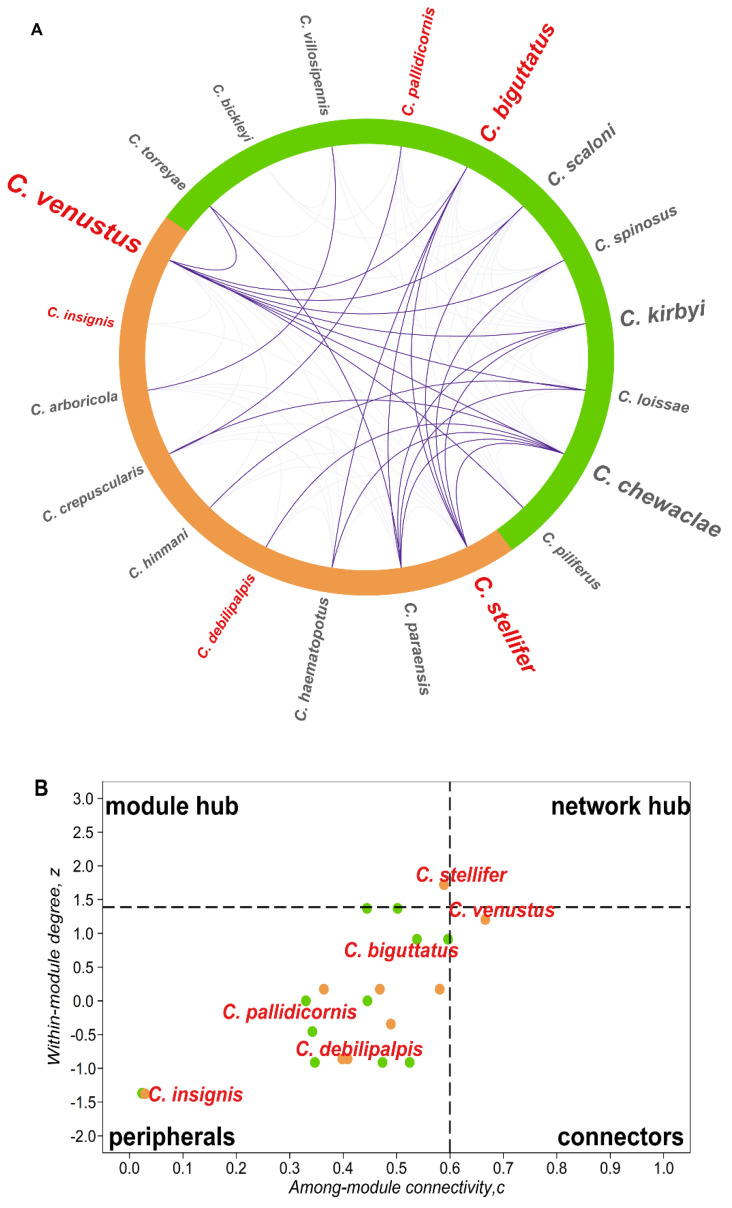
Co-occurrence *Culicoides* network highlights seasonal compartments connected by vector species: weekly co-occurrence circular network maps and species roles. (**A**) Circular network: species are arranged in the two modules nesting seasons occurrence (Figure S1.4; summer–fall: orange; winter–spring: blue). Co-occurrence interactions between modules are drawn by a stronger opaque links pattern (violet) than within module interactions (transparent violet). Label size is relative to species degree (Table S1.4); vectors are red printed and plotted with circleplot [108]. (**B**) Species roles in the co-occurrence network sustaining and connecting modular configuration are shown in dimensions defined by *z* (within-module degree) and *c* (among-module connectivity) scores (Table S1.4). Dot colors follow modules in **A**, but only vector species are labeled. Dashed lines: threshold value for *z* (horizontal line: 1.39) and for *c* (vertical line: 0.6).

**Figure 7:**
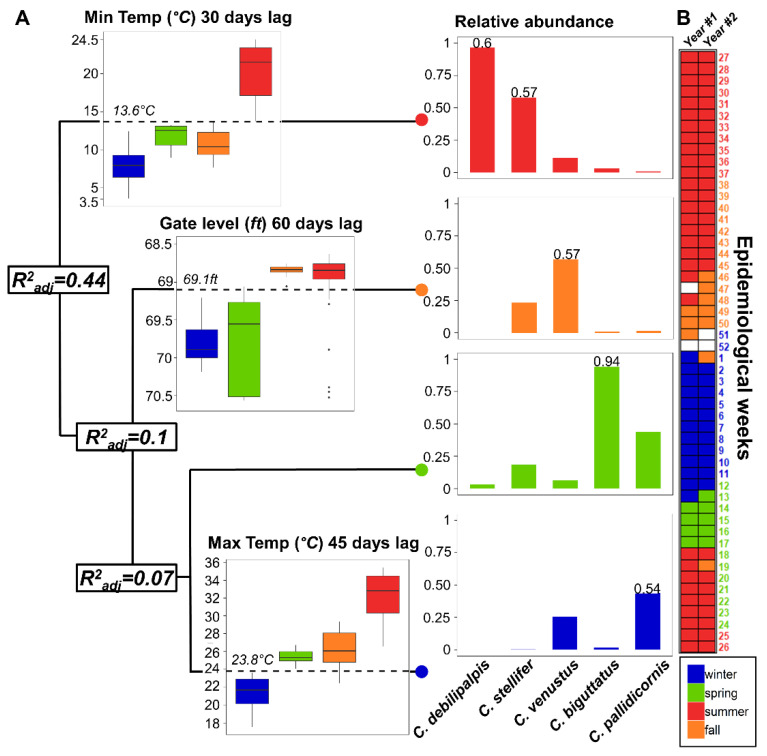
*Culicoides* vector subassemblage is time-partitioned through a hierarchy of environmental thresholds. (**A**) The regression tree displays the clustering process for the dissimilarity of vector subassemblage among sampling weeks achieved through a sequence of environmental thresholds. Branch splitting gives place to nodes (colored dots; cluster of epidemiological weeks) and a new branch in a way to predict the amount of variation explained at each step (R^2^_adj_). Distribution of environmental variables at the clusters level are plotted in a box-chart, and threshold value is printed. Moreover, vector relative abundance distribution is presented in bar-charts, and significant indicator species value is labeled. (**B**) Matching between clusters and calendar is represented by a time-line matrix (Year #1: July 2015–June 2016; Year #2: July 2016–June 2017). Cells are filled as a cluster and week labels colored as calendar season. Empty cells represent weeks without sampling.

The *Culicoides* community was active year-round and connected by the pattern of vector co-occurrence (Figure 6A). The weekly occurrence network supported the two season modules (M = 0.24; *p <* 0.01): winter–spring and summer–fall (Figure 6A). Species displayed most of their interactions with species within the same module (Figure 6A; lighter shade links), e.g., winter prevalent species and spring prevalent species overlapped their activity during several weeks. In addition, species did not co-occur equally. For example, species occurrence and degree (*C_d_*) were heterogeneously distributed as *C. venustus* (*C_d_* = 15), *C. stellifer* (*C_d_* = 13) and *C. haematopotus* (*C_d_* = 7) and occupied 80% of surveyed weeks in both years, while the remainder were recorded in less than 40% of the weeks (Figure S1.4, Table S1.4). A total of 90% of species also co-occurred with at least one species out of its module (Figure 7A; dark shade links). This result emphasizes how connectivity among modules expanded co-occurrence beyond traditional seasons (Figure 6B). *Culicoides venustus* played a connector role among the two modules by overlapping time, environment and HD transmission role between species. In addition, *C. stellifer*, as a module hub, connected the summer–fall module. All other *Culicoides* were peripheral, as they co-occurred more commonly with species displaying similar seasonality. Midge activity was not detected in five weeks in 2016 and just one week in 2017 (January and February, Table S1.5), when average minimum temperatures (2.16 ± 0.38 °C) were below the winter months’ first quartile (Table S1.3, Figure S1.5). Finally, occurrence length and co-occurrence pattern among *Culicoides* linked vector persistence across the entire year, producing a suitable scenario to locally support the maintenance of arbovirus transmission.

### 3.4. Environmental Thresholds in the Temporal Dynamic of the Vector Assemblage

Clustering analysis defined weather thresholds as an important driver of vector assemblage (Figure 7A). The resulting tree predicted 61% of the variation in the abundance with four informative nodes (Root node error = 0.46 (47.03/101); CV_error_ = 0.67; SE = 0.09) and three environmental dimensions that were defined by stream condition and temperature lags (Figure 7). Precipitation was not selected by MVRT as a relevant source to predict variation in the vector assemblage. Nodes or clusters represented groups of independent epidemiological weeks which were allocated between nodes in the two sampling years. First, a minimum temperature of 13.6 °C (T_min30_) was able to explain 44% of the variation in the vector assemblage (Figure 7A) and was explained by a warm node that spanned between the last half of the spring to the first half of fall. This node absorbed 97% of the abundance for *C. debilipalpis* (id = 0.6; *p* < 0.01) and 58% for *C. stellifer* (id = 0.57; *p* < 0.01; Figure 7A). Second, a 10% improvement in the clustering was explained by stream flow level (gauge_60_ = 69.1 ft), at the end of fall, and represented 57% of *C. venustus* abundance (id = 0.57; *p* < 0.01). Finally, maximum temperature (T_max45_ = 23.8 °C) explained 7% of the vector temporal dynamic by a node related with some early spring weeks (97% in *C. biguttatus* abundance; id = 0.97; *p* < 0.01) and a consistent winter node (43% in *C. pallidicornis* abundance; id = 0.54; *p* < 0.01). Additionally, epidemiological weeks mismatched with calendar season after node allocation (Figure 7B). Phenological dynamics of the vector community were dominated by arrays of species associated with defined ecological thresholds that were not congruent with calendar seasons.

## 4. Discussion

The community assembly in *Culicoides* species followed several independent temporal profiles that were linked by environmental variables, neutral drivers and historical process. Importantly, the seasonal dynamics of *Culicoides* relied on a biannual phenology, highlighting that overimposed calendar schemes do not necessary fit the community responses [44]. For instance, northern Florida falls within the temperate domain of southeastern USA making it tempting to analyze communities using a four-season regime. The ordination pattern of *Culicoides* that we observed, however, is more consistent with a biannual seasonality structure commonly seen in subtropical and tropical biomes [39,109,110,111]. This pattern of subtropical seasonality was consistently detected with our different modeling approaches (diversity composition, diversity structure, co-occurrence network and vector subassemblage partitioning among environmental thresholds).

### 4.1. Seasonality of Culicoides β-Diversity

The winter–spring and summer–fall modules of species co-occurrence and niche partitioning of the vector assemblage coincide with the dry–wet scheme of the Oceanic and Tropical-Subtropical climate regimes of Florida [39,111]. Our data indicate that *Culicoides* species have a sequential and overlapping emergence that ensures temporal connectivity within the vector community. The early season, cold-tolerant species, transitional species and warm weather species may provide seasonal persistence of pathogen vectors, as supported by the harmonic overlap of species demographies in our models which identified recruitment waves of vector assemblages rather than any single dominant vector species. Multivoltinism in subtropical–tropical *Culicoides* [48] should increase overlap. Even though large pulses during the summer–fall months were observed (Figure S1.6, Table S1.5), we did not find biotic interactions, supporting the assertion that species coexistence relies on competitive processes [112].

Both temperature and stream flow shaped the likelihood of *Culicoides* species co-occurrence (Figure 7). Gerry and Mullens [113] identified temperature rather than rainfall as a driver of seasonal dynamics of *Culicoides* in temperate regions. However, temperature usually interacts with rainfall in *Culicoides* dynamics [39,110,114,115]. Therefore, the distribution of the thermal niche between species subassemblages appears to contribute to the stability of BTV–EHDV transmission. The distribution of the thermal niche among species in the subassemblages is relevant (Figure 3 and Figure 7, Figure S1.3) as both longevity and larval developmental times in *Culicoides* species are modulated by the species thermal sensitivity [116,117,118]. We propose that the fluctuations in temperature associated with the temporal change in *Culicoides* composition could also be affecting the overall load of EHDV–BTV transmission. For instance, the outcome of the virus–*Culicoides* interaction in the episystem (i.e., vector capacity, [23]) is sensitive to temperature because abundance, survival (demography) and extrinsic incubation period (virogenesis) covary with the temperature a *Culicoides* species experiences [116,119,120,121,122]. Consequently, temperature has a strong net effect on *Culicoides-*borne *Orbivirus*.

The finding that stream dynamics and temperature drive seasonality in *Culicoides β-*diversity, but precipitation does not, indicates that nonprecipitation sources of water shape the moisture conditions driving larval development and population pulses of adults [37,123,124,125]. In the panhandle of Florida, water from a superficial aquifer emerges in the slopes as seepages and runs off into streams, and clay soils cause surface pooling [51]. Consequently, the aquifer and stream interact with the *Culicoides’* niche. For instance, *C. haematopotus, C. stellifer* and *C. venustus* develop at stream borders, puddles and seepage habitats, respectively [126], and moisture and nutrient availability in these habitats are subject to the dynamics of stream flooding and aquifer surfacing [51].

The lack of significance of rainfall is surprising, given the intrinsic link between water and semiaquatic breeding sites of *Culicoides* [127] and the previously documented association between precipitation and *Culicoides* seasonality [39,110,114,115,128]. Nevertheless, precipitation was not related to *Culicoides* abundance in some studies [36,114,129,130,131]. For instance, rainfall might be patchily distributed, but this spatial variability is expected to underestimate daily precipitation rather than the lag explored here. Importantly, our finding does not exclude the effect of rainfall on *Culicoides* but helps to explain how weather may constrain each population, community and functional group differently. As such, phenology proved less sensitive to rainfall, specifically, than to other measures of hydrology (stream flow).

The co-effect of temperature and stream flow configure the timing for community seasonal dynamics given the entire *Culicoides* species deploys a range of responses to moisture and temperature. For example, the least diverse assemblage (RDA_1_) arose early in the epidemiological calendar (Figure 2B), was mainly composed of winter–early spring species and was highly correlated with all of the selected stream and temperature variables (Figure S1.3); meanwhile, the most diverse assemblage (RDA_2_) arose later in the calendar, and was composed of a mixture of spring and summer–fall species exclusively correlated with temperature. We hypothesize that flooding early in the wet season resupplies the soil moisture lost during dry fall–winter months and nutrients drained by precipitation and recurrent flooding events during the wet season. In addition, it is interesting how the dynamics of the soil moisture and HD are linked. In the panhandle, the pattern of HD occurrence is supported by the enzootic stability hypothesis [69] as the relationship between high BTV/EHDV transmission rate and herd immunity [27] is associated with low mortality [69]. Christensen et al. [132] shows that the increase in drought severity has a protective effect on HD mortality occurrence within the counties in the top 20% of wetland cover and bellow 30° latitude, which define our study location. Therefore, drought-flooding dynamics may lead to the enzootic stability of HD in association with *Culicoides* community change.

Our analysis showed that niche and neutral drivers partitioned equally *Culicoides β*-diversity seasonality, not from variability in flying activity and null noise in field data, as previously suggested [36,47,50,131,133]. Neutral drivers could be overestimated, as other variables (i.e., wind patterns, humidity, atmospheric pressure and daylight duration) were not measured but are expected to correlate with the environmental conditions explored here. It seems less likely that confounding neutral drivers, unexplored environmental layers and noisy data are major drivers of *Culicoides β*-diversity seasonality. Importantly, this so-called black box of “unexplained” variation in populations and community of *Culicoides* are susceptible to being biologically explained after stochastic processes are considered.

### 4.2. Seasonal Culicoides Diversity Structure

Organization in *Culicoides* diversity aligns with both nested and modular temporal topologies, relying upon the persistence of a few species (Figure 5 and Figure 6, Figure S1.4). For example, we observed that 90% of *Culicoides* species occurred in weeks when species of another seasonal module were present (Figure 5 and Figure 6A). As such, *Culicoides* richness and composition are temporally nested (Figure 5) and may follow similar ecological rules. It also means that changes in diversity depend on how *Culicoides* species are lost over time but not by the replacement of some species by others. Community climax occurred in the spring weeks (Figure 4) even though only 14% of the species displayed unique springtime phenology. In temperate regions, univoltine and rare species drive the high *Culicoides* richness in spring months [48]. Some *Culicoides* species might be ecologically redundant, as species exhibit temporal co-occurrence that significantly overlaps niche conditions, and they seem to share abundant resources [40,134]. Overall, this indicates that community structure is not organized by temperate calendar seasons, and the unified neutral theory of biodiversity is suitable to explain *Culicoides* diversity.

### 4.3. Persistence of Vector-Borne Pathogen in the Ecosystems Depends on Environmental Stability

Northern Florida provides a permissive environment to sustain the year-round transmission of *Culicoides*-borne *Orbivirus* as shown by vector persistence in the region. The longest observed period without host-seeking activity (5 weeks) does not signify arrested larval development, since *C. pallidicornis* adult emergence was continuous during winter weeks (Figure S1.6). The absence of *Culicoides* in traps during this period could be due to biological shifts in circadian rhythms, with normally nocturnal species shifting their host-seeking activity to warmer daylight hours [135,136,137]. *Culicoides venustus* and *C. pallidicornis* can be exposed to EHDV infectious blood meals as seroconversion and viremic resident white-tailed deer have been recorded in northern Florida during the winter. Alternatively, vertical and horizontal transmission, in addition to the persistence host infection, may facilitate the maintenance of EHDV–BTV during winter [26,138,139,140]. Vector recruitment is nearly continuous for HD transmission, and our data suggest that *C. venustus* provides a link between fall and spring months. Nonetheless, no *Culicoides* species behaved as a network hub connecting all species and modules. Additional data on the infection prevalence of BTV and EHDV in *Culicoides* will help to understand the link between the continuous vector recruitment and the year-round persistence of HD. Operations such as animal movement and trade during winter months should proceed with caution as the vector-free period does not occur in northern Florida.

### 4.4. Is Culicoides β-Diversity Operating under Wider Temporal Scales?

Three wider temporal scales (≥12 months, Figure 2C) revealed that *Culicoides* communities experience annual and cyclic-periodic oscillation that could extend beyond two years. We propose that different processes operate between the wider scales. Here, we describe the annual variation at the *Culicoides* community level for the first time in Florida (Figure 2; RDA_2,3_) and that it is strongly related to the fluctuation in the environmental conditions. Importantly, the annual incidence of HD also correlates with environmental variables, especially drought severity, weather conditions and land cover change in the USA [25,132,133]. We speculate that the annual temporal scale is related to how species recruitment and the local environment are shaped by global-scale weather patterns. Both phases of El Niño Southern Oscillation (ENSO) took place during the study [141] and coincide with the observed effect of the temperature and stream level on annual *Culicoides* dynamics (Figure 3A, Figure S1.3; RDA_2_). Interestingly, ENSO has been previously implicated in *Culicoides-*borne episystems [142] where in southeastern USA, HD morbidity has occurred every 2–3 years [26,68,143].

When periodicity in community composition shows innate signals (RDA_1_; Figure 2C,D, Figure S1.3) long-term biogeographical processes under wider spatial scales are expected to impact *β-*diversity. This emphasizes the trade-off between detrending data for satisfying mathematical assumptions and the risk of obscuring ecological patterns [11,87]. Altogether, *Culicoides β*-diversity agrees well with the space–time duality predictions [9]. In general, the wider scales are more dynamic and better suited to environmental filtering than seasonality. Moreover, temporal patterns in HD occurrence are strongly dependent on the spatial hierarchy since between years and periods, HD is nested at county and biogeographical scales [132,133]. Thus, we suggest that regional patterns in HD can also be predicted by surveying local scenarios of *Orbivirus* transmission in *Culicoides* community data.

## 5. Conclusions

The dynamics in the *Culicoides* community followed a subtropical seasonal regime driven by the atmospheric temperature and ground water early in the season and by temperature later in the season. The merit of this study relies on how we allowed the system to inform the phenological pattern instead of imposing a classical temperate seasonal scheme. Neutral drivers were relevant as niche drivers. Conversely, the relevance of the ecological drift on assembling was lacking, even though unexplained variation was related to noisy data niche-based studies.

The management of *Culicoides*-borne *Orbivirus* risk depends on disrupting the pathogen niche through seasonally-appropriate and targeted activities. In northern Florida, the disrupting of larval breeding during spring could be a valuable tool to interrupt HD transmission; but the use of stations for insecticide and repellent application may not be economically viable given the persistence and dispersal ability of *Culicoides*. Field evaluations are needed to assess these statements.

## Supplementary Materials

The following are available online at https://www.mdpi.com/1999-4915/12/9/931/s1. Supplementary File 1. Table S1.1: Temporal scales and their AEM eigenfunction associated in *Culicoides* composition change; Table S1.2: Species of *Culicoides* related with community change composition at each temporal scale; Table S1.3: Weeks without *Culicoides* activity recorded and associated daily meteorology; Table S1.4: Temporal network of co-occurring *Culicoides*; Table S1.5: Monthly, year and total weekly *Culicoides* abundance, density and presence; Figure S1.1: Map of the study area and light traps distribution in Gadsden County, Florida; Figure S1.2: *Culicoides* composition time autocorrelation by multivariate Mantel’s correlogram; Figure S1.3: Environmental constraints on *Culicoides β*-diversity temporal profiles; Figure S1.4: *Culicoides* season occurrence in the two modules derived from the weekly co-occurrence network; Figure S1.5: Daily temperature between December 20th and April 20th (2015–2016 and 2016–2017) in Tallahassee airport, Florida; Figure S1.6: Aging of the vector subassemblage: (A) spread of parity among seasons, (B) parity seasonal trend; Figure S1.7. Vector assemblage and seasonality sampling coverage: sample completeness curve. Supplementary File 2: *Culicoides β*-diversity spatiotemporal dynamics; Supplementary File 3: *Culicoides* abundance and environmental data, R code script.
