# Supplementary material for: Tracking Community Timing: Pattern and Determinants of Seasonality in Culicoides (Diptera: Ceratopogonidae) in Northern Florida
Source: Viruses. 2020 Aug 25;12(9):931. doi: 10.3390/v12090931 (PMC7552033; doi:10.3390/v12090931)
Supplement: Supplementary file 1 [file viruses-12-00931-s001.zip › viruses-863910-for conversion-suppl/Supplementary 1_viruses-863910[July24].pdf]

Supplementary File 1

In: Tracking community timing: pattern and determinants of seasonality in *Culicoides* (Diptera: Ceratopogonidae) in northern Florida.

Agustín I. Quaglia [1,*], Erik M. Blosser [2], Bethany L. McGregor [1,2], Alfred E. Runkel IV [1], Kristin E. Sloyer [1], Dinesh Erram [1], Samantha M. Wisely [3] and Nathan D. Burkett-Cadena[1]

[1] Florida Medical Entomology Laboratory, University of Florida, 200 9th St. SE, Vero Beach, FL 32962, USA;
[2] United States Department of Agriculture, 1515 College Ave., Manhattan, Kansas, 66502, USA;
[3] Department of Wildlife Ecology and Conservation, University of Florida, 110 Newins-Ziegler Hall, Gainesville, Florida, 32611, USA
* Correspondence: aquaglia@ufl.edu; Tel.: +01-772-226-6644 (AIQ)

Table S1.1. Temporal scales and their *AEM* eigenfunction associated in *Culicoides* composition change. Subset of *AEM* eigenfunction selected after forward selection of the positive (50 *AEMs*) and negative (50 *AEMs*) temporal spectral decomposition.

Temporal Scale	*Fvalue; df; p*	R^2_{adj}	*AEM eigenfuctions*	RDA_{axis}	*F-value; df; p*	*Week range*
Wide	20.33; 4; 0.001	0.44	1, 3, 4, 5	1	2.79; 1; 0.047	~82.72
				2	14.65; 1; 0.001	58.71
				3	63.28; 1; 0.001	53
Medium	3.32; 7; 0.001	0.14	7, 8, 9 ,11, 12, 13, 17	1	10.08; 1; 0.002	23.99 - 29
				2	6.57; 1; 0.042	25.14 - 26.86
Small	0.88; 2; 0.496	NA	38, 42	NA	NA	NA
Negative	0.21; 50; 1	NA	0	NA	NA	NA

Over 52,178 caught individuals in 101 weeks, an assemblage of 21 out 29 species were suitable to perform *β*-diversity temporal profile analysis (total abundance >10; *n*=52,136; 0.08% of the full dataset). *F-value, df* and *p*: Fisher test value, degree of freedom and *p* with permutation analysis (*n*=1,000). R^2_{adj}: Adjusted determination coefficient for each temporal scale explaining change in the community composition. *AEM* eigenfunctions: id. number means an increasing order in the temporal span pattern. RDA_{axis}: significant axis in redundancy analysis after regressing with the *AEM* eigenfunctions subset (permutation analysis, *n*=1,000). Week range: is the minimum and maximum oscillation durations (weeks) in the linear combinators scores temporal fluctuations for a given RDA_{axis}.

Table S1.2. Species of *Culicoides* related with community change composition at each temporal scale. Spearman rank correlation between *RDA* linear combinators scores and species abundance.

Taxonomic group	***Species***	***Larval habitat***	***Scale***	***RDA$_{axis}$***	**|*Rho*|**
Oecacta	*C. stellifer*	ground-dwelling	Wide	1	0.03
				2	0.72*
				3	0.26*
			Medium	1	0.05
				2	0.38*
Silvaticulicoides	*C. biguttatus*	ground-dwelling	Wide	1	0.15
				2	0.12
				3	0.61*
			Medium	1	0.1
				2	0.5*
	C. loisae	ground-dwelling	Wide	1	0.11
				2	0.02
				3	0.29*
			Medium	1	0.04
				2	0.11
	C. spinosus	ground-dwelling	Wide	1	0.16
				2	0.15
				3	0.45*
			Medium	1	0.08
				2	0.58*
Haematomyidium	*C. debilipalpis*	ground-dwelling	Wide	1	0.14
				2	0.69*
				3	0.2*
			Medium	1	0.08
				2	0.06
	C. paraensis	ground-dwelling	Wide	1	0.15
				2	0.38*
				3	0.12
			Medium	1	0.02
				2	0.01
	C. torreyae	unknow	Wide	1	0.13
				2	0.12
				3	0.19
			Medium	1	0.07
				2	0.02

Taxonomic groups: *Culicoides* subgeneric classification in Borkent [1]; Larval habitat according to [2,3]; |*Rho*|: absolute Spearman rank score, *: $p \leq 0.05$

Table S1.2. Continue.

Taxonomic group	*Species*	*Larval habitat*	*Scale*	*RDA$_{axis}$*	ǀ*Rho*ǀ
Diphaomyia	*C. haematopotus*	ground-dwelling	Wide	1	0.08
				2	0.56*
				3	0.2*
			Medium	1	0.06
				2	0.42*
Hoffamnia	*C. insignis*	ground-dwelling	Wide	1	0.25*
				2	0.08
				3	0.35*
			Medium	1	0.26*
				2	0.14
	C. venustus	ground-dwelling	Wide	1	0.22*
				2	0.38*
				3	0.19
			Medium	1	0.07
				2	0.43*
Stonei	*C. pallidicornis*	ground-dwelling	Wide	1	0.18
				2	0.46*
				3	0.6*
			Medium	1	0.28*
				2	0.33*
Piliferus	*C. bickleyi*	ground-dwelling	Wide	1	0.15
				2	0.42*
				3	0.42*
			Medium	1	0.26*
				2	0.11
	C. chewaclae	unknow	Wide	1	0.18
				2	0.02
				3	0.23*
			Medium	1	0.07
				2	0.29*
	C. kirbyi	unknow	Wide	1	0.09
				2	0.03
				3	0.35*
			Medium	1	0.15
				2	0.34*

Table S1.2. Continue.

Taxonomic group	*Species*	*Larval habitat*	*Scale*	*RDA$_{axis}$*	∣*Rho*∣
Piliferus	*C. piliferus*	ground-dwelling	Wide	1	0.1
				2	0.03
				3	0.08
			Medium	1	0.2*
				2	0.42*
	C. scaloni	ground-dwelling	Wide	1	0.15
				2	0.15
				3	0.38*
			Medium	1	0.01
				2	0.41*
Amossovia	*C. arboricola*	tree holes	Wide	1	0.1
				2	0.43*
				3	0.26*
			Medium	1	0.04
				2	0.08
	C. villosipennis	tree holes	Wide	1	0.07
				2	0.18*
				3	0.17
			Medium	1	0.12
				2	0.01
Beltranmyia	*C. crepuscularis*	ground-dwelling	Wide	1	0.21*
				2	0.1
				3	0.09
			Medium	1	0.04
				2	0.11
Drymodesmyia	*C. hinmani*	tree holes	Wide	1	0.09
				2	0.41*
				3	0.02
			Medium	1	0.12
				2	0.09
Monoculicoides	*C. variipennis*	ground-dwelling	Wide	1	0.08
				2	0.11
				3	0.21*
			Medium	1	0.05
				2	0.24*

Table S1.3. Weeks without *Culicoides* activity recorded and associated daily meteorology.

Season	*Epidemiological Week*	*Date*	*Precipitation (mm³)*	*T°min (C°)*	*T°max (C°)*
Winter	1	2016-01-04	139.2	0	28.3
	2	2016-01-11	3	-1.1	18.3
	3	2016-01-18*	0	1.7	14.4
	4	2016-01-25	0	-2.2	18.9
	6	2016-02-08	0	0	13.9
		2017-02-06	0	3.9	25
		$\bar{x} \pm sd$	23.7 ± 56.59	0.38 ± 2.16	19.8 ± 5.77

*Data took from Tallahassee airport meteorological station (USW00093805) as non-available data was registered in the nearest meteorological station (USC00087429).

Table S1.4. Temporal network of co-occurring *Culicoides*. Weekly occurrence and species level network metrics.

	Total	*Spring*	*Summer*	*Fall*	*Winter*	*Degree*	*z*	*c*
C. arboricola	33.33	44.83	61.54	12.5	0	6	0.17	0.36
C. bickleyi	25	51.72	0	0	52.94	3	-1.37	0.02
C. biguttatus	22.92	72.41	3.85	0	0	12	0.91	0.54
C. chewaclae	5.21	17.24	0	0	0	14	0.91	0.60
C. crepuscularis	16.67	20.69	11.54	16.67	17.65	6	-0.34	0.49
C. debilipalpis	40.62	44.83	80.77	20.83	0	4	-0.86	0.40
C. haematopotus	80.21	86.21	100	83.33	35.29	7	0.17	0.47
C. hinmani	10.42	3.45	34.62	0	0	4	-0.86	0.41
C. insignis	16.67	10.34	0	41.67	17.65	2	-1.38	0.03
C. kirbyi	7.29	24.14	0	0	0	12	1.37	0.50
C. loisae	11.46	27.59	7.69	0	5.88	7	-0.91	0.52
C. pallidicornis	34.38	79.31	0	0	58.82	7	0.00	0.33
C. paraensis	16.67	13.79	38.46	8.33	0	10	0.17	0.58
C. piliferus	14.58	27.59	0	25	0	6	-0.46	0.34
C. scanloni	11.46	34.48	0	0	5.88	11	1.37	0.44
C. spinosus	30.21	72.41	3.85	20.83	11.76	8	0.00	0.45
C. stellifer	80.21	100	100	83.33	11.76	13	1.72	0.59
C. torreyae	5.21	13.79	3.85	0	0	6	-0.91	0.47
C. villosipennis	2.08	6.9	0	0	0	5	-0.91	0.35
C. venustus	94.79	96.55	100	100	76.47	15	1.20	0.67

Occurrence week-species matrix holds *Culicoides* with more than 5 individuals caught and recorded at least in 5% of the weeks (~5 weeks out of 95). Total, Spring, Summer, Fall, Winter: proportion (%) of weeks when species were recorder; Degree: number of occurring partners for a *Culicoides*; *z*: within-module degree; *c*: among-module connectivity. Summer-Fall module: orange; Winter-Spring module: green.

Figure S1.1. Map of the study area and light traps distribution in Gadsden County, Florida.

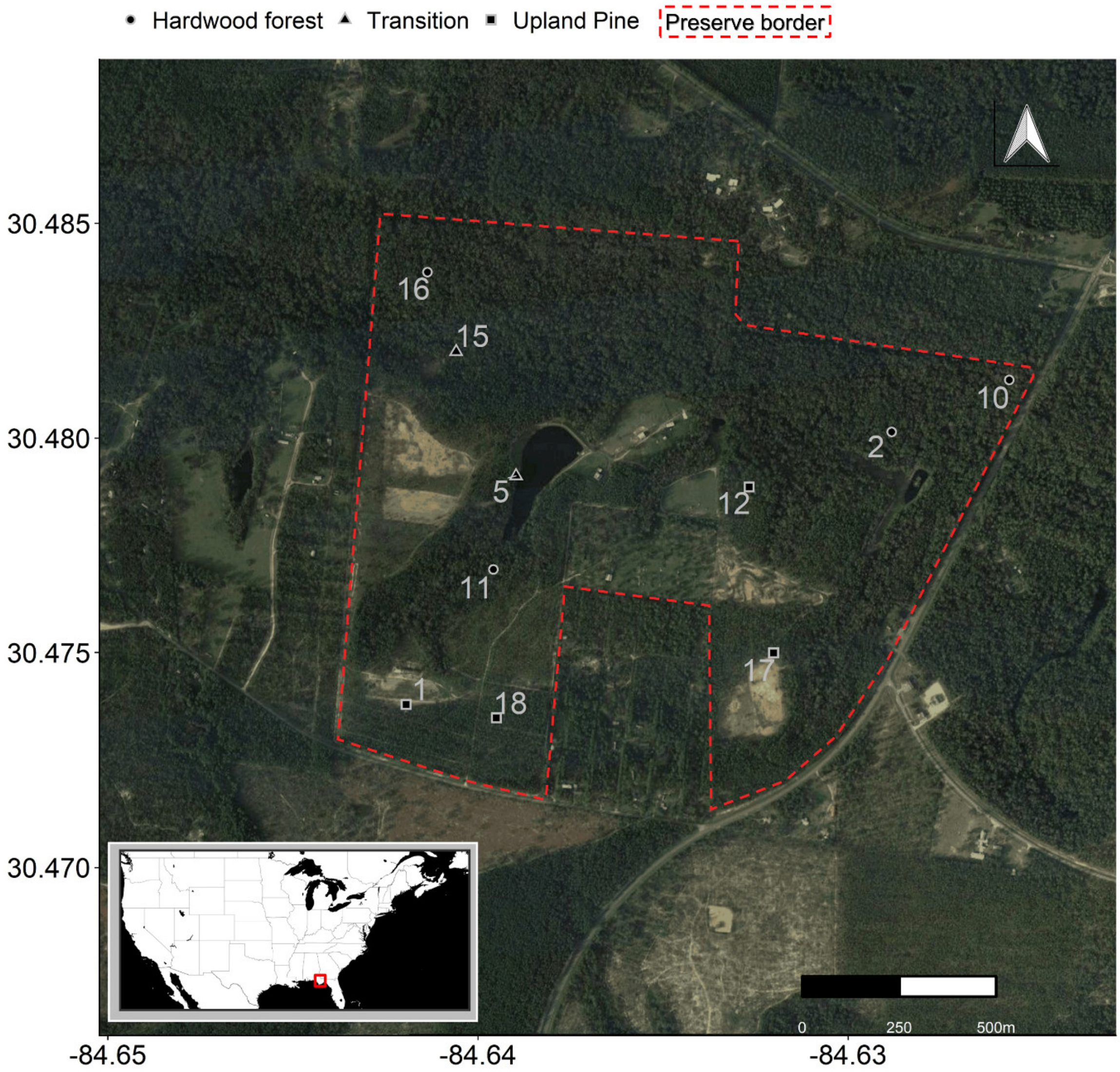

Ten traps were located within the major habitats in the preserve (180 ha): bottomland hardwoods (4traps), upland pine stands (4 traps) and the transition between both (2 traps). Two deer pens housed roughly half of the property's White-tailed deer. The study site was mapped with ggmap[4] from Google Earth.

Figure S1.2. *Culicoides* composition time autocorrelation by multivariate Mantel's correlogram. Minimum number of days between independent observations.

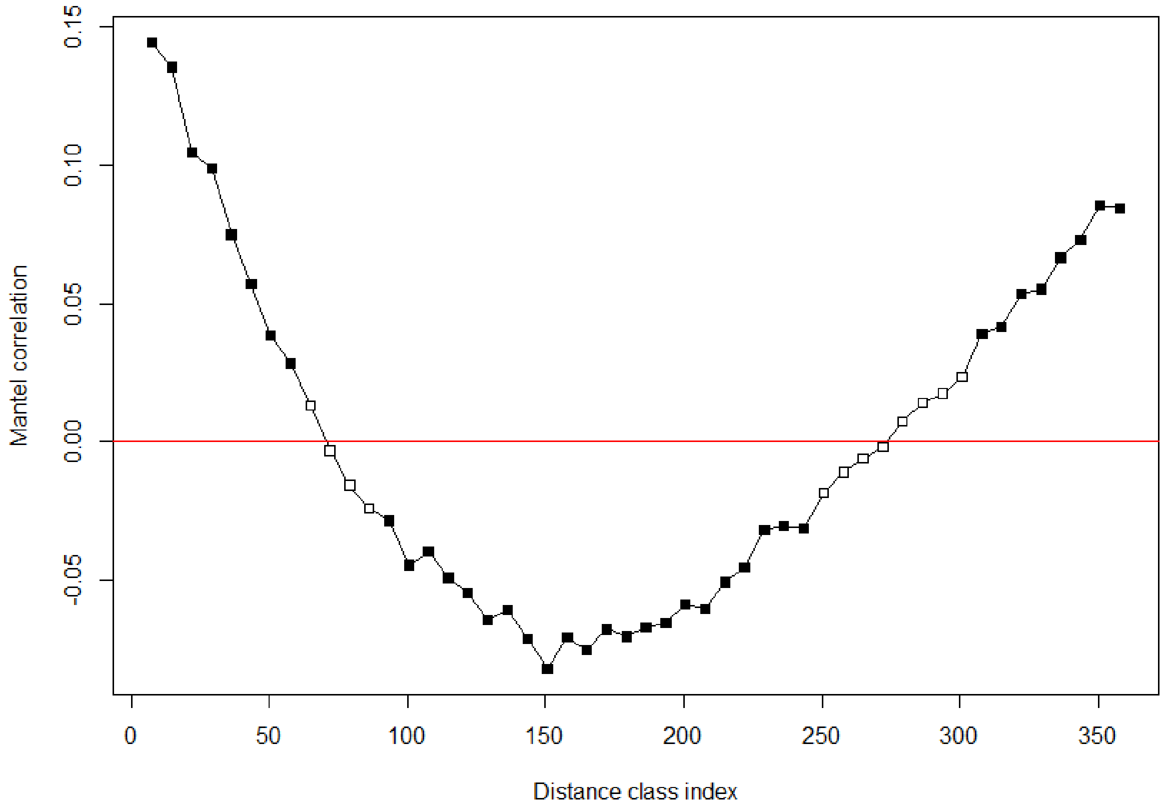

Distance class index: distance (days) between sampling dates. Above red line shows positive Mantel correlation values, indeed positive autocorrelation. Black filled squares identify significant Mantel correlation values (p <0.05) after multiple testing with Holm's method.

Figure S1.3. Environmental constraints on *Culicoides* β-diversity temporal profiles.

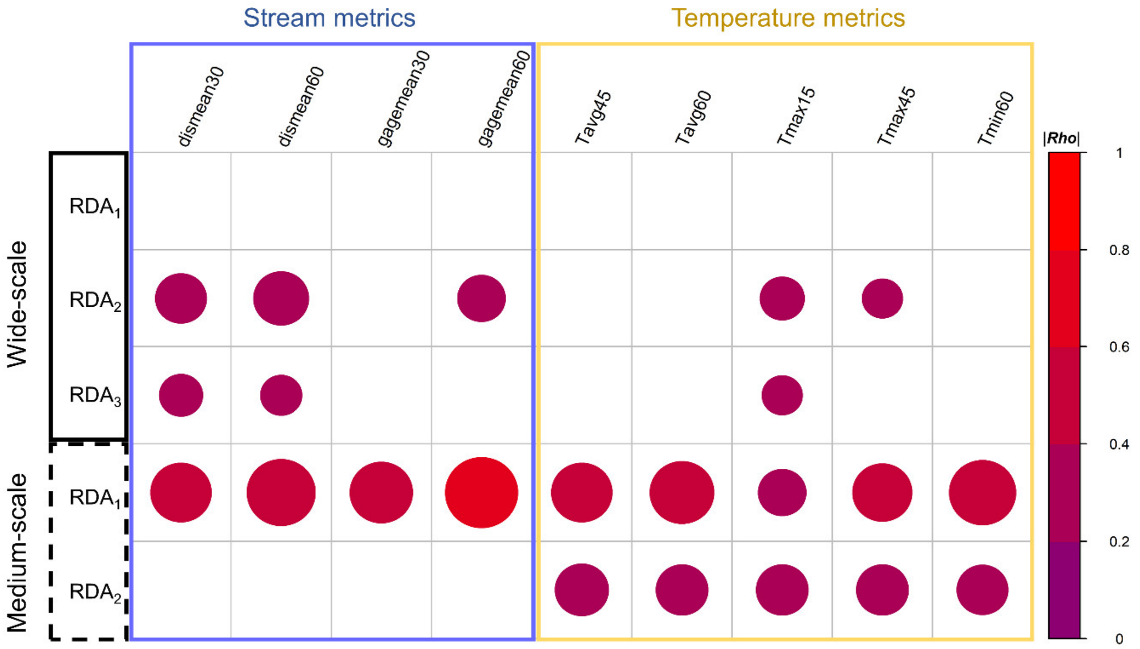

Spearman rank correlation between *RDA* linear combinators scores and stream-temperature. Table S1.1 detail the temporal dimensions for the *RDA* profiles. Stream and temperatures variables represent the selected subset of environmental variables in Figure 3: Stream submodel was comprised by the mean gate height (*ft*) and discharge flow (*ft*^{3}/*second*) at 30- and 60-days lags from community observation; Temperature submodel comprises minimum (60 days lag), maximum (15-45 days lag) and mean (45-60 days lag) atmospheric temperature (°C). |*Rho*|: significant absolute Spearman rank scores ($p \leq 0.05$). Scores magnitude is represented by circle color and size.

Figure S1.4. *Culicoides* season occurrence in the two modules derived from the weekly co-occurrence network.

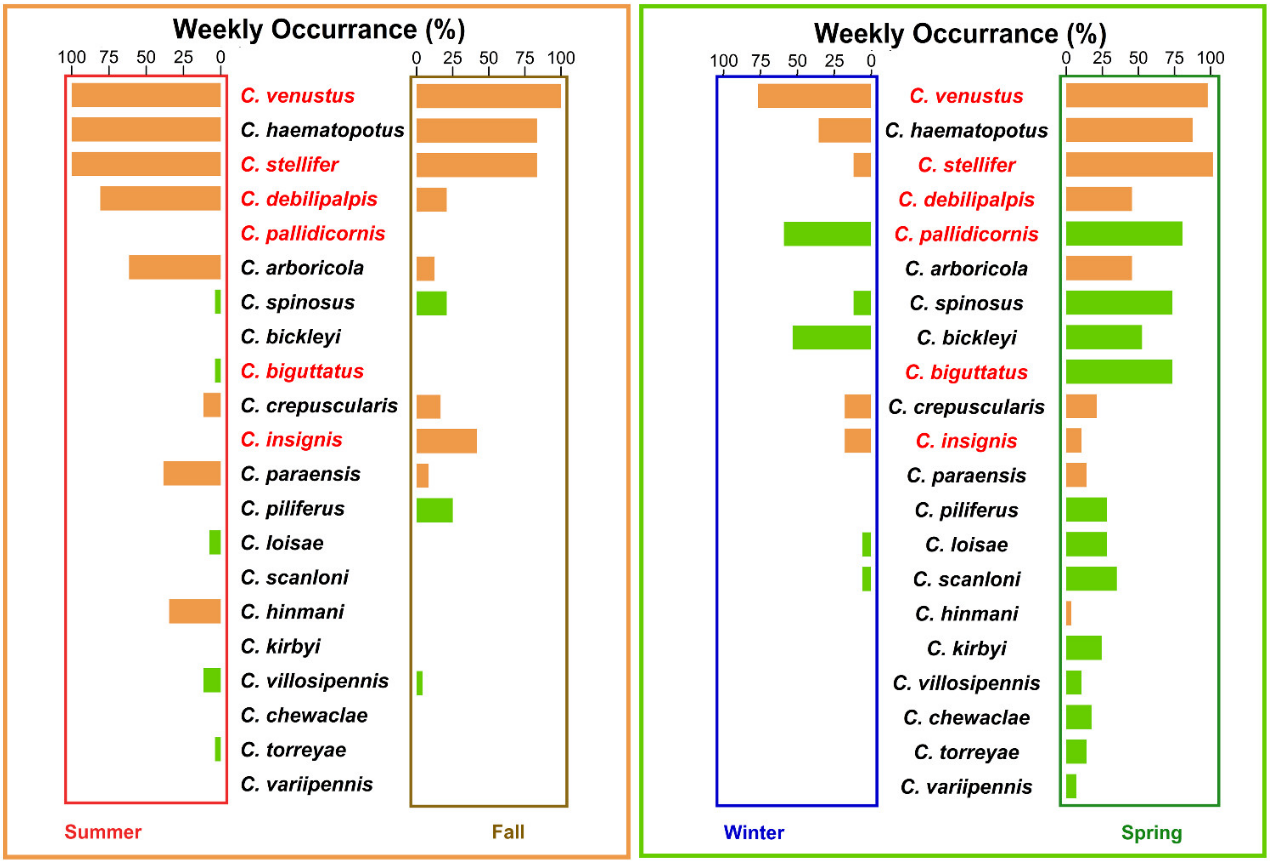

Largest squares fallow the code color from network co-occurrence modules (Figure 6 main manuscript). Colors in the bars represent the species module's affiliation and vector were red labeled. Bar charts extend to the subset of species suitable for the analysis (*Culicoides* with more than 5 individuals and recorded at least in 5% of the weeks, ~5 weeks out of 95).

Figure S1.5. Daily temperature between December 20th- April 20th (2015-2016 and 2016-2017) in Tallahassee airport, Florida.

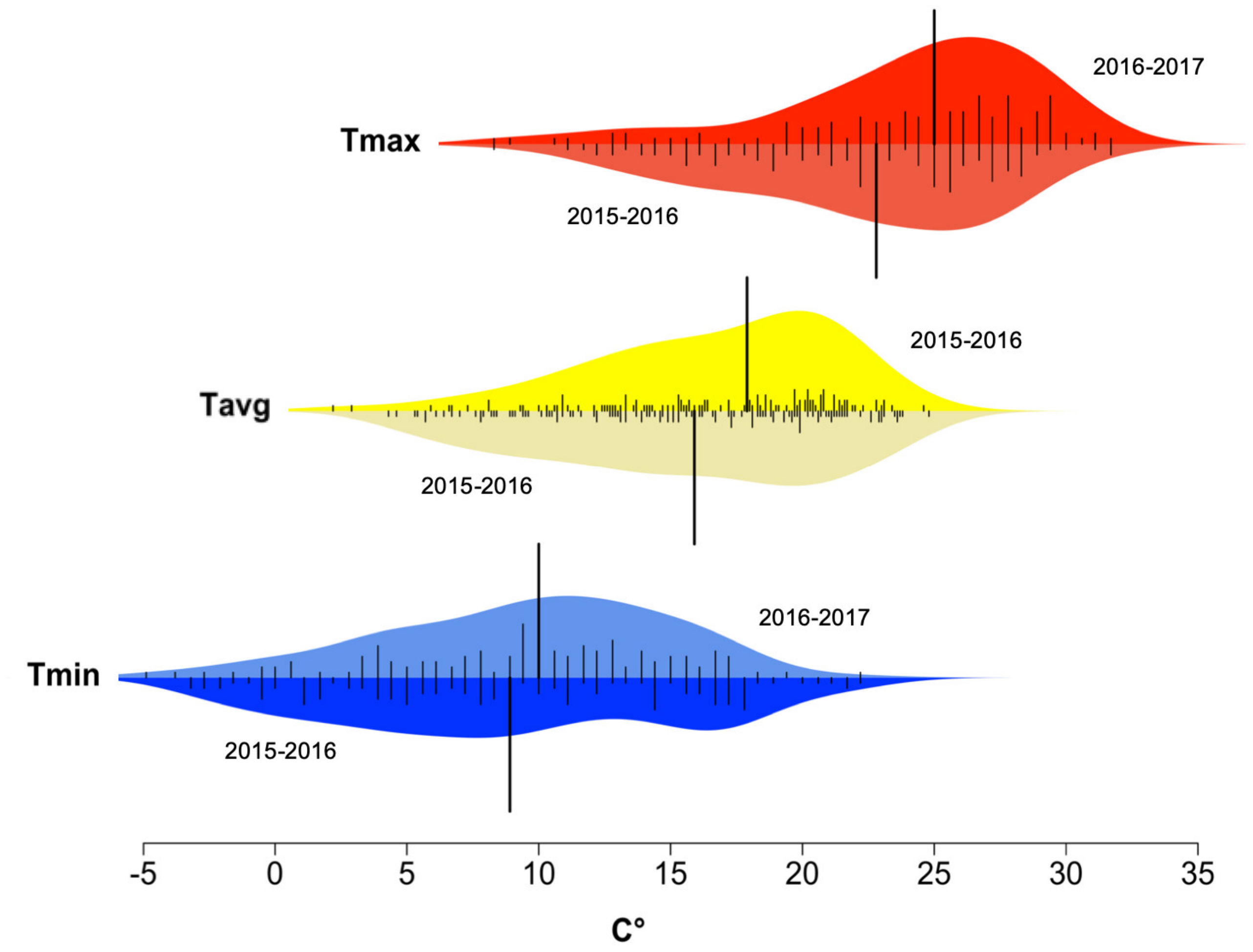

Tmin: minimum temperature; *Tavg*= average temperature; *Tmax*: maximum temperature. Long bar: median temperature. Tallahassee airport meteorological station (USW00093805).

Figure S1.6. Aging of the vector subassemblage: A) Spread of parity among seasons. B) Parity seasonal trend.

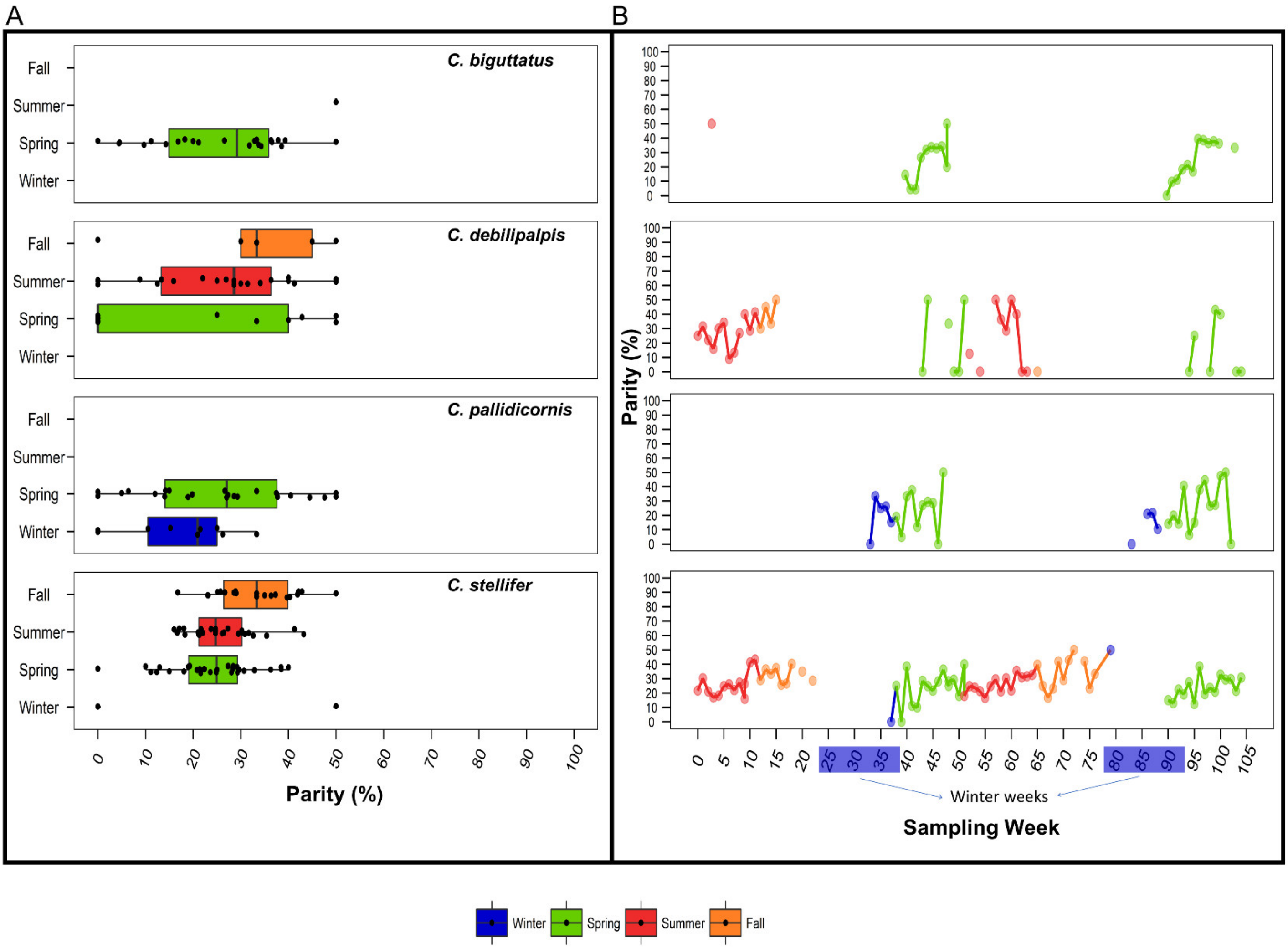

Parity: number of parous out of parous plus nulliparous. Purple weeks represented weeks were Culicoides abundance ≅ 0

Figure S1.7. Vector assemblage and seasonality sampling coverage: sample completeness curve.

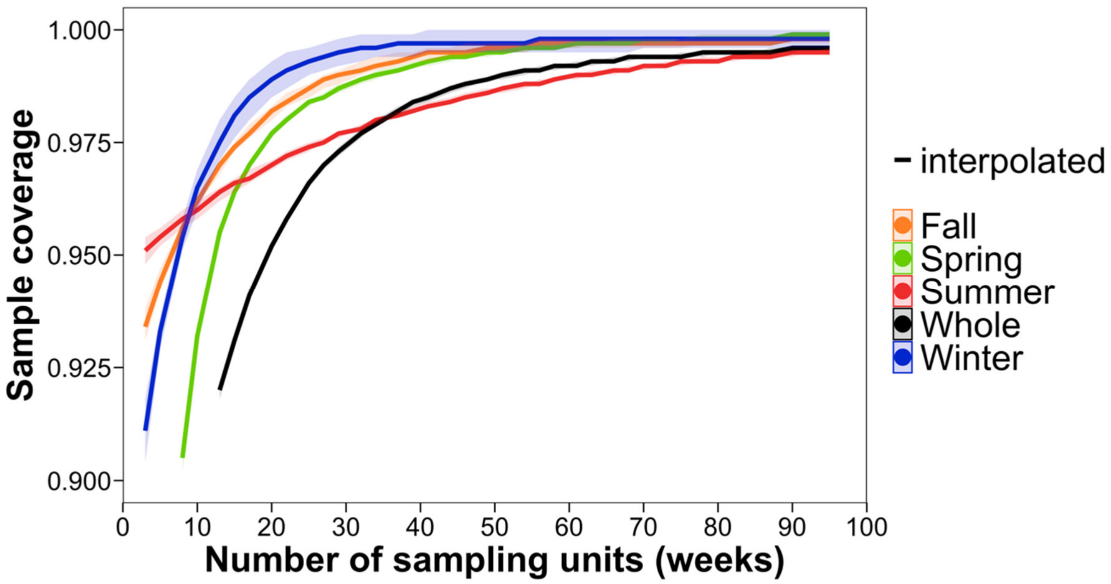

Table S1.5. Monthly, year and total weekly *Culicoides* abundance, density and presence.

Species	Year	*January* week=3; active=1.9-1.7; trap=10			*February* week=4; active=1.7-1.9; trap=10			*March* week=5-4; active=1.8-1.9; trap=10			*April* week=4; active=1.8-1.9; trap=10		
		**Density**	**Abund**	**Presence**	**Density**	**Abund**	**Presence**	**Density**	**Abund**	**Presence**	**Density**	**Abund**	**Presence**
C. arboricola[βɪ]	I	0(0)	0	0(0)	0(0)	0	0(0)	0(0)	0	0(0)	0.05(0.05)	2	25(1)
	II	0(0)	0	0(0)	0(0)	0	0(0)	0.1(0.05)	4	25(1)	0.1(0.06)	4	75(3)
C. bickleyi[βɪ]	I	0(0)	0	0(0)	0.2(0.11)	8	75(3)	0.08(0.04)	4	40(2)	0.38(0.18)	15	75(3)
	II	0(0)	0	0(0)	0.65(0.2)	26	75(3)	1.45(0.53)	58	75(3)	0.68(0.25)	27	100(4)
C. biguttatus[βɪ]	I	0(0)	0	0(0)	0(0)	0	0(0)	0(0)	0	0(0)	20.55(8.32)	822	100(4)
	II	0(0)	0	0(0)	0(0)	0	0(0)	6.5(2.35)	260	50(2)	26.5(5.68)	1,060	100(4)
C. chewaclae[βɪ]	I	0(0)	0	0(0)	0(0)	0	0(0)	0(0)	0	0(0)	0(0)	0	0(0)
	II	0(0)	0	0(0)	0(0)	0	0(0)	0(0)	0	0(0)	0.08(0.06)	3	50(2)
C. crepuscularis[βɪ]	I	0(0)	0	0(0)	0(0)	0	0(0)	0(0)	0	0(0)	0.08(0.04)	3	50(2)
	II	0(0)	0	0(0)	0.1(0.06)	4	50(2)	0.03(0.03)	1	25(1)	0.05(0.03)	2	50(2)
C. debilipalpis[βɪ]	I	0(0)	0	0(0)	0(0)	0	0(0)	0(0)	0	0(0)	0.03(0.03)	1	25(1)
	II	0(0)	0	0(0)	0(0)	0	0(0)	0(0)	0	0(0)	0.1(0.06)	4	50(2)
C. haematopotus[βɪ]	I	0(0)	0	0(0)	0(0)	0	0(0)	0.04(0.03)	2	40(2)	7.25(5.32)	290	75(3)
	II	0.07(0.05)	2	33(1)	0.23(0.09)	9	50(2)	2.43(1.05)	97	75(3)	18.08(6.19)	723	100(4)
C. hinmani[βɪ]	I	0(0)	0	0(0)	0(0)	0	0(0)	0(0)	0	0(0)	0(0)	0	0(0)
	II	0(0)	0	0(0)	0(0)	0	0(0)	0(0)	0	0(0)	0(0)	0	0(0)
C. insignis[βɪ]	I	0(0)	0	0(0)	0(0)	0	0(0)	0(0)	0	0(0)	0(0)	0	0(0)
	II	0.03(0.03)	1	33(1)	0(0)	0	0(0)	0.05(0.05)	2	25(1)	0(0)	0	0(0)
C. kirbyi[βɪ]	I	0(0)	0	0(0)	0(0)	0	0(0)	0(0)	0	0(0)	0(0)	0	0(0)
	II	0(0)	0	0(0)	0(0)	0	0(0)	0(0)	0	0(0)	0.08(0.08)	3	25(1)
C. loisae[βɪ]	I	0(0)	0	0(0)	0(0)	0	0(0)	0(0)	0	0(0)	0(0)	0	0(0)
	II	0(0)	0	0(0)	0.03(0.03)	1	25(1)	0(0)	0	0(0)	0.08(0.04)	3	50(2)
C. pallidicornis[βɪ]	I	0(0)	0	0(0)	0.08(0.04)	3	50(2)	21.62(13.53)	1,081	100(5)	4.15(1.83)	166	100(4)
	II	0.03(0.03)	1	33(1)	2.43(1.34)	97	75(3)	19.25(8.21)	770	75(3)	78.55(29.76)	3,142	100(4)
C. paraensis[βɪ]	I	0(0)	0	0(0)	0(0)	0	0(0)	0(0)	0	0(0)	0(0)	0	0(0)
	II	0(0)	0	0(0)	0(0)	0	0(0)	0(0)	0	0(0)	0.05(0.03)	2	25(1)
C. piliferus[βɪ]	I	0(0)	0	0(0)	0(0)	0	0(0)	0(0)	0	0(0)	0(0)	0	0(0)
	II	0(0)	0	0(0)	0(0)	0	0(0)	0(0)	0	0(0)	0.2(0.11)	8	50(2)
C. pusillus	I	0(0)	0	0(0)	0(0)	0	0(0)	0(0)	0	0(0)	0(0)	0	0(0)
	II	0(0)	0	0(0)	0(0)	0	0(0)	0(0)	0	0(0)	0(0)	0	0(0)
C. scanloni[βɪ]	I	0(0)	0	0(0)	0(0)	0	0(0)	0(0)	0	0(0)	0.08(0.06)	3	50(2)
	II	0(0)	0	0(0)	0.03(0.03)	1	25(1)	0(0)	0	0(0)	0.83(0.35)	33	75(3)
C. spinosus[βɪ]	I	0(0)	0	0(0)	0(0)	0	0(0)	0.22(0.18)	11	40(2)	1.6(0.41)	64	100(4)
	II	0(0)	0	0(0)	0.03(0.03)	1	25(1)	3.25(1.55)	130	50(2)	3.1(0.7)	124	100(4)

β: Culicoides included in *β*-diversity temporal profile analysis (detailed in Statistical analysis section); *ɪ*: *Culicoides* included in weekly co-occurrence network (detailed in Statistical analysis section); Year: July 2015 to July 2016 (*I*), July 2016 to July 2017 (*II*); week: number of weeks; active: mean number of nights for a week that a trap was active; trap: number of trap-site; Density (*se*): Mean number of midge captured per trap/week (standard error); Abund: total number of midge captured; Presence: species weekly occurrence (number of week recorded); Rel.abund: species relative abundance out total midge caught at each year and total of period a total (%), <0.01 relative abundance less than 0.01%.

Table S1.5. Monthly, year and total weekly *Culicoides* abundance, density and presence. Continue

Species	Year	*January* week=3; active=1.9-1.7; trap=10			*February* week=4; active=1.7-1.9; trap=10			*March* week=5-4; active=1.8-1.9; trap=10			*April* week=4; active=1.8-1.9; trap=10		
		**Density**	**Abund**	**Presence**	**Density**	**Abund**	**Presence**	**Density**	**Abund**	**Presence**	**Density**	**Abund**	**Presence**
C. stellifer$^{\beta'}$	I	0(0)	0	0(0)	0(0)	0	0(0)	0.12(0.06)	6	60(3)	7.6(2.65)	304	100(4)
	II	0.03(0.03)	1	33(1)	0(0)	0	0(0)	1.5(0.56)	60	50(2)	24.98(6.96)	999	100(4)
C. torreyae$^{\beta'}$	I	0(0)	0	0(0)	0(0)	0	0(0)	0(0)	0	0(0)	0(0)	0	0(0)
	II	0(0)	0	0(0)	0(0)	0	0(0)	0(0)	0	0(0)	0(0)	0	0(0)
C. variipennis$^{\beta}$	I	0(0)	0	0(0)	0(0)	0	0(0)	0(0)	0	0(0)	0.2(0.18)	8	50(2)
	II	0(0)	0	0(0)	0(0)	0	0(0)	0(0)	0	0(0)	0(0)	0	0(0)
C. venustus$^{\beta'}$	I	0(0)	0	0(0)	0.13(0.08)	5	25(1)	0.78(0.4)	39	80(4)	1.05(0.3)	42	100(4)
	II	0.17(0.08)	5	67(2)	1.88(0.59)	75	75(3)	3.35(0.95)	134	100(4)	4.98(1.3)	199	100(4)
C. villosipennis$^{\beta'}$	I	0(0)	0	0(0)	0(0)	0	0(0)	0(0)	0	0(0)	0(0)	0	0(0)
	II	0(0)	0	0(0)	0(0)	0	0(0)	0(0)	0	0(0)	0(0)	0	0(0)
C. alachua/ sanguisuga	I	0(0)	0	0(0)	0(0)	0	0(0)	0(0)	0	0(0)	0(0)	0	0(0)
	II	0(0)	0	0(0)	0.03(0.03)	1	25(1)	0.03(0.03)	1	25(1)	0.03(0.03)	1	25(1)
C. baueri	I	0(0)	0	0(0)	0(0)	0	0(0)	0(0)	0	0(0)	0(0)	0	0(0)
	II	0(0)	0	0(0)	0(0)	0	0(0)	0(0)	0	0(0)	0(0)	0	0(0)
C. beckae	I	0(0)	0	0(0)	0(0)	0	0(0)	0(0)	0	0(0)	0(0)	0	0(0)
	II	0(0)	0	0(0)	0(0)	0	0(0)	0(0)	0	0(0)	0(0)	0	0(0)
C. furens	I	0(0)	0	0(0)	0(0)	0	0(0)	0(0)	0	0(0)	0.03(0.03)	1	25(1)
	II	0(0)	0	0(0)	0(0)	0	0(0)	0(0)	0	0(0)	0.03(0.03)	1	25(1)
C. guttipennis	I	0(0)	0	0(0)	0(0)	0	0(0)	0(0)	0	0(0)	0(0)	0	0(0)
	II	0(0)	0	0(0)	0(0)	0	0(0)	0(0)	0	0(0)	0.03(0.03)	1	25(1)
C. nanus	I	0(0)	0	0(0)	0(0)	0	0(0)	0(0)	0	0(0)	0(0)	0	0(0)
	II	0(0)	0	0(0)	0(0)	0	0(0)	0(0)	0	0(0)	0(0)	0	0(0)
C. ousairani	I	0(0)	0	0(0)	0(0)	0	0(0)	0(0)	0	0(0)	0.03(0.03)	1	25(1)
	II	0(0)	0	0(0)	0(0)	0	0(0)	0(0)	0	0(0)	0.03(0.03)	1	25(1)
C. spp.	I	0(0)	0	0(0)	0(0)	0	0(0)	0(0)	0	0(0)	0(0)	0	0(0)
	II	0(0)	0	0(0)	0(0)	0	0(0)	0(0)	0	0(0)	0(0)	0	0(0)
**Total: Year-Month**	I	0(0)	0	-	1.6(0.64)	16	-	114.3(77.87)	1,143	-	172.2(56.13)	1,722	-
	II	1(0.33)	10	-	21.5(7.01)	215	-	151.7(43.9)	1,517	-	634(215.53)	6,340	-
**Total: Month**		0.17(0.07)	10	-	2.89(0.81)	231	-	29.56(8.72)	2,660	-	100.78(20.32)	8,062	-

Table S1.5. Monthly, year and total weekly *Culicoides* abundance, density and presence. Continue

Species	Year	***May*** week=5; active=1.9-1.5; trap=10			***June*** week=4; active=1.7-1.9; trap=10			***July*** week=5-4; active=1.7; trap=10			***August*** week=5; active=1.7-2; trap=10		
		**Density**	**Abund**	**Presence**	**Density**	**Abund**	**Presence**	**Density**	**Abund**	**Presence**	**Density**	**Abund**	**Presence**
C. arboricola[β']	I	0.04(0.03)	2	40(2)	0.08(0.06)	3	50(2)	0.4(0.13)	20	80(4)	0.84(0.39)	42	100(5)
	II	0.16(0.12)	8	60(3)	0.13(0.08)	5	75(3)	0(0)	0	0(0)	0.04(0.03)	2	40(2)
C. bickleyi[β']	I	0.18(0.1)	9	60(3)	0(0)	0	0(0)	0(0)	0	0(0)	0(0)	0	0(0)
	II	0.12(0.05)	6	40(2)	0.03(0.03)	1	25(1)	0(0)	0	0(0)	0(0)	0	0(0)
C. biguttatus[β']	I	16.08(6.23)	804	100(5)	0(0)	0	0(0)	0.02(0.02)	1	20(1)	0(0)	0	0(0)
	II	8.18(3.37)	409	100(5)	0.1(0.06)	4	25(1)	0(0)	0	0(0)	0(0)	0	0(0)
C. chewaclae[β']	I	0(0)	0	0(0)	0(0)	0	0(0)	0(0)	0	0(0)	0(0)	0	0(0)
	II	0.3(0.2)	15	60(3)	0(0)	0	0(0)	0(0)	0	0(0)	0(0)	0	0(0)
C. crepuscularis[β']	I	0(0)	0	0(0)	0(0)	0	0(0)	0.08(0.04)	4	40(2)	0.04(0.04)	2	20(1)
	II	0.04(0.03)	2	40(2)	0(0)	0	0(0)	0(0)	0	0(0)	0(0)	0	0(0)
C. debilipalpis[β']	I	0.06(0.03)	3	40(2)	0.25(0.1)	10	100(4)	2.2(0.48)	110	100(5)	2.3(0.41)	115	100(5)
	II	0.16(0.07)	8	60(3)	0.1(0.05)	4	50(2)	0.05(0.05)	2	25(1)	0.36(0.11)	18	100(5)
C. haematopotus[β']	I	16.2(8.18)	810	100(5)	6.88(3.02)	275	100(4)	12.24(3.98)	612	100(5)	16.42(11.5)	821	100(5)
	II	14.14(7.16)	707	100(5)	2.7(1.06)	108	100(4)	4.98(3.67)	199	100(4)	6.56(5.17)	328	100(5)
C. hinmani[β']	I	0(0)	0	0(0)	0.03(0.03)	1	25(1)	0.02(0.02)	1	20(1)	0.2(0.11)	10	60(3)
	II	0(0)	0	0(0)	0(0)	0	0(0)	0.03(0.03)	1	25(1)	0.06(0.03)	3	60(3)
C. insignis[β']	I	0.02(0.02)	1	20(1)	0(0)	0	0(0)	0(0)	0	0(0)	0(0)	0	0(0)
	II	0(0)	0	0(0)	0.03(0.03)	1	25(1)	0(0)	0	0(0)	0(0)	0	0(0)
C. kirbyi[β']	I	0.16(0.09)	8	60(3)	0(0)	0	0(0)	0(0)	0	0(0)	0(0)	0	0(0)
	II	0.6(0.31)	30	60(3)	0(0)	0	0(0)	0(0)	0	0(0)	0(0)	0	0(0)
C. loisae[β']	I	0.04(0.03)	2	40(2)	0.03(0.03)	1	25(1)	0(0)	0	0(0)	0.02(0.02)	1	20(1)
	II	0.08(0.05)	4	40(2)	0.03(0.03)	1	25(1)	0(0)	0	0(0)	0(0)	0	0(0)
C. pallidicornis[β']	I	1.96(0.93)	98	80(4)	0(0)	0	0(0)	0(0)	0	0(0)	0(0)	0	0(0)
	II	11.42(4.25)	571	100(5)	0.05(0.03)	2	50(2)	0(0)	0	0(0)	0(0)	0	0(0)
C. paraensis[β']	I	0(0)	0	0(0)	0(0)	0	0(0)	0.32(0.11)	16	80(4)	0.28(0.09)	14	100(5)
	II	0.04(0.03)	2	40(2)	0.03(0.03)	1	25(1)	0(0)	0	0(0)	0(0)	0	0(0)
C. piliferus[β']	I	0.22(0.16)	11	60(3)	0(0)	0	0(0)	0(0)	0	0(0)	0(0)	0	0(0)
	II	2.1(1.44)	105	60(3)	0(0)	0	0(0)	0(0)	0	0(0)	0(0)	0	0(0)
C. pusillus	I	0(0)	0	0(0)	0(0)	0	0(0)	0(0)	0	0(0)	0(0)	0	0(0)
	II	0(0)	0	0(0)	0(0)	0	0(0)	0(0)	0	0(0)	0(0)	0	0(0)
C. scanloni[β']	I	0.3(0.16)	15	40(2)	0(0)	0	0(0)	0(0)	0	0(0)	0(0)	0	0(0)
	II	0.32(0.14)	16	60(3)	0(0)	0	0(0)	0(0)	0	0(0)	0(0)	0	0(0)
C. spinosus[β']	I	0.74(0.22)	37	100(5)	0(0)	0	0(0)	0(0)	0	0(0)	0(0)	0	0(0)
	II	0.98(0.34)	49	80(4)	0.03(0.03)	1	25(1)	0(0)	0	0(0)	0.02(0.02)	1	20(1)

Table S1.5. Monthly, year and total weekly *Culicoides* abundance, density and presence. Continue

Species	Year	May week=5; active=1.9-1.5; trap=10			June week=4; active=1.7-1.9; trap=10			July week=5-4; active=1.7; trap=10			August week=5; active=1.7-2; trap=10		
		**Density**	**Abund**	**Presence**	**Density**	**Abund**	**Presence**	**Density**	**Abund**	**Presence**	**Density**	**Abund**	**Presence**
C. stellifer$^{\beta I}$	I	83.52(23.6)	4,176	100(5)	43.83(12.69)	1,753	100(4)	47.04(9.75)	2,352	100(5)	56.44(19.34)	2,822	100(5)
	II	64.5(16.12)	3,225	100(5)	16.15(4.53)	646	100(4)	17.8(6.15)	712	100(4)	28.8(8.94)	1,440	100(5)
C. torreyae$^{\beta I}$	I	0(0)	0	0(0)	0(0)	0	0(0)	0(0)	0	0(0)	0(0)	0	0(0)
	II	0.14(0.08)	7	60(3)	0.05(0.05)	2	25(1)	0(0)	0	0(0)	0(0)	0	0(0)
C. variipennis$^{\beta}$	I	0(0)	0	0(0)	0(0)	0	0(0)	0(0)	0	0(0)	0(0)	0	0(0)
	II	0(0)	0	0(0)	0(0)	0	0(0)	0(0)	0	0(0)	0(0)	0	0(0)
C. venustus$^{\beta I}$	I	5.6(1.5)	280	100(5)	3.18(0.86)	127	100(4)	1.6(0.44)	80	100(5)	1.48(0.34)	74	100(5)
	II	4.66(1.71)	233	100(5)	1(0.28)	40	100(4)	2.45(0.92)	98	100(4)	3.24(0.84)	162	100(5)
C. villosipennis$^{\beta I}$	I	0.12(0.12)	6	20(1)	0(0)	0	0(0)	0.06(0.03)	3	40(2)	0.02(0.02)	1	20(1)
	II	0.04(0.04)	2	20(1)	0.03(0.03)	1	25(1)	0(0)	0	0(0)	0(0)	0	0(0)
C. alachua/ sanguisuga	I	0.04(0.03)	2	20(1)	0(0)	0	0(0)	0(0)	0	0(0)	0(0)	0	0(0)
	II	0(0)	0	0(0)	0(0)	0	0(0)	0(0)	0	0(0)	0(0)	0	0(0)
C. baueri	I	0.02(0.02)	1	20(1)	0(0)	0	0(0)	0.02(0.02)	1	20(1)	0.02(0.02)	1	20(1)
	II	0.1(0.06)	5	40(2)	0(0)	0	0(0)	0(0)	0	0(0)	0.02(0.02)	1	20(1)
C. beckae	I	0(0)	0	0(0)	0(0)	0	0(0)	0.04(0.03)	2	40(2)	0.02(0.02)	1	20(1)
	II	0(0)	0	0(0)	0(0)	0	0(0)	0(0)	0	0(0)	0(0)	0	0(0)
C. furens	I	0(0)	0	0(0)	0(0)	0	0(0)	0(0)	0	0(0)	0(0)	0	0(0)
	II	0.1(0.05)	5	40(2)	0(0)	0	0(0)	0(0)	0	0(0)	0(0)	0	0(0)
C. guttipennis	I	0(0)	0	0(0)	0(0)	0	0(0)	0(0)	0	0(0)	0.02(0.02)	1	20(1)
	II	0(0)	0	0(0)	0.08(0.04)	3	50(2)	0(0)	0	0(0)	0(0)	0	0(0)
C. nanus	I	0(0)	0	0(0)	0(0)	0	0(0)	0.04(0.03)	2	20(1)	0.02(0.02)	1	20(1)
	II	0.02(0.02)	1	20(1)	0(0)	0	0(0)	0(0)	0	0(0)	0(0)	0	0(0)
C. ousairani	I	0(0)	0	0(0)	0(0)	0	0(0)	0(0)	0	0(0)	0.02(0.02)	1	20(1)
	II	0(0)	0	0(0)	0(0)	0	0(0)	0(0)	0	0(0)	0(0)	0	0(0)
C. spp.	I	0(0)	0	0(0)	0.03(0.03)	1	25(1)	0(0)	0	0(0)	0(0)	0	0(0)
	II	0(0)	0	0(0)	0(0)	0	0(0)	0(0)	0	0(0)	0(0)	0	0(0)
**Total: Year-Month**	I	626.5(208.12)	6,265	-	217.1(66.81)	2,171	-	320.4(75.12)	3,204	-	390.7(151.88)	3,907	-
	II	541(138.49)	5,410	-	82(22.8)	820	-	101.2(41.21)	1,012	-	195.5(76.43)	1,955	-
**Total: Month**		116.75(20.64)	11,675	-	37.39(7.81)	2,991	-	46.84(8.24)	4,216	-	58.62(13.74)	5,862	-

Table S1.5. Monthly, year and total weekly *Culicoides* abundance, density and presence. Continue

Species	Year	*September* week=4; active=1.7-1.9; trap=10			*October* week=4-5; active=1.7-1.9; trap=10			*November* week=3-4; active=1.8-1.7; trap=10			*December* week=3-2; active=1.6-1.9; trap=10		
		**Density**	**Abund**	**Presence**	**Density**	**Abund**	**Presence**	**Density**	**Abund**	**Presence**	**Density**	**Abund**	**Presence**
C. arboricola$^{\beta'}$	I	0.8(0.43)	32	100(4)	0.05(0.05)	2	25(1)	0(0)	0	0(0)	0(0)	0	0(0)
	II	0.13(0.13)	5	25(1)	0(0)	0	0(0)	0(0)	0	0(0)	0(0)	0	0(0)
C. bickleyi$^{\beta'}$	I	0(0)	0	0(0)	0(0)	0	0(0)	0(0)	0	0(0)	0(0)	0	0(0)
	II	0(0)	0	0(0)	0(0)	0	0(0)	0(0)	0	0(0)	0(0)	0	0(0)
C. biguttatus$^{\beta'}$	I	0(0)	0	0(0)	0(0)	0	0(0)	0(0)	0	0(0)	0(0)	0	0(0)
	II	0(0)	0	0(0)	0(0)	0	0(0)	0(0)	0	0(0)	0(0)	0	0(0)
C. chewaclae$^{\beta'}$	I	0(0)	0	0(0)	0(0)	0	0(0)	0(0)	0	0(0)	0(0)	0	0(0)
	II	0(0)	0	0(0)	0(0)	0	0(0)	0(0)	0	0(0)	0(0)	0	0(0)
C. crepuscularis$^{\beta'}$	I	0(0)	0	0(0)	0.03(0.03)	1	25(1)	0(0)	0	0(0)	0(0)	0	0(0)
	II	0(0)	0	0(0)	0(0)	0	0(0)	0.05(0.03)	2	50(2)	0.05(0.05)	1	50(1)
C. debilipalpis$^{\beta'}$	I	1.55(0.91)	62	100(4)	0.08(0.04)	3	50(2)	0(0)	0	0(0)	0(0)	0	0(0)
	II	0.1(0.05)	4	75(3)	0(0)	0	0(0)	0(0)	0	0(0)	0(0)	0	0(0)
C. haematopotus$^{\beta'}$	I	13.35(4.32)	534	100(4)	12.7(7.98)	508	100(4)	4.53(3.56)	136	67(2)	0.1(0.07)	3	33(1)
	II	6.1(3.49)	244	100(4)	10.3(4.82)	515	100(5)	2.88(1.5)	115	75(3)	0.2(0.12)	4	100(2)
C. hinmani$^{\beta'}$	I	0.08(0.06)	3	25(1)	0(0)	0	0(0)	0(0)	0	0(0)	0(0)	0	0(0)
	II	0(0)	0	0(0)	0(0)	0	0(0)	0(0)	0	0(0)	0(0)	0	0(0)
C. insignis$^{\beta'}$	I	0(0)	0	0(0)	0.5(0.48)	20	25(1)	0.2(0.09)	6	33(1)	0(0)	0	0(0)
	II	0.05(0.03)	2	25(1)	0.2(0.1)	10	60(3)	0.4(0.27)	16	25(1)	0.5(0.2)	10	100(2)
C. kirbyi$^{\beta'}$	I	0(0)	0	0(0)	0(0)	0	0(0)	0(0)	0	0(0)	0(0)	0	0(0)
	II	0(0)	0	0(0)	0(0)	0	0(0)	0(0)	0	0(0)	0(0)	0	0(0)
C. loisae$^{\beta'}$	I	0.03(0.03)	1	25(1)	0(0)	0	0(0)	0(0)	0	0(0)	0(0)	0	0(0)
	II	0(0)	0	0(0)	0(0)	0	0(0)	0(0)	0	0(0)	0(0)	0	0(0)
C. pallidicornis$^{\beta'}$	I	0(0)	0	0(0)	0(0)	0	0(0)	0(0)	0	0(0)	0(0)	0	0(0)
	II	0(0)	0	0(0)	0(0)	0	0(0)	0(0)	0	0(0)	0(0)	0	0(0)
C. paraensis$^{\beta'}$	I	0.05(0.03)	2	50(2)	0.05(0.03)	2	25(1)	0(0)	0	0(0)	0(0)	0	0(0)
	II	0(0)	0	0(0)	0(0)	0	0(0)	0(0)	0	0(0)	0(0)	0	0(0)
C. piliferus$^{\beta'}$	I	0(0)	0	0(0)	0.13(0.09)	5	50(2)	0.03(0.03)	1	33(1)	0(0)	0	0(0)
	II	0(0)	0	0(0)	0.04(0.04)	2	20(1)	0.08(0.06)	3	50(2)	0(0)	0	0(0)
C. pusillus	I	0(0)	0	0(0)	0(0)	0	0(0)	0(0)	0	0(0)	0(0)	0	0(0)
	II	0(0)	0	0(0)	0(0)	0	0(0)	0(0)	0	0(0)	0.05(0.05)	1	50(1)
C. scanloni$^{\beta'}$	I	0(0)	0	0(0)	0(0)	0	0(0)	0(0)	0	0(0)	0(0)	0	0(0)
	II	0(0)	0	0(0)	0(0)	0	0(0)	0(0)	0	0(0)	0(0)	0	0(0)
C. spinosus$^{\beta'}$	I	0.03(0.03)	1	25(1)	0.03(0.03)	1	25(1)	0(0)	0	0(0)	0(0)	0	0(0)
	II	0(0)	0	0(0)	0.08(0.05)	4	60(3)	0(0)	0	0(0)	0(0)	0	0(0)

Table S1.5. Monthly, year and total weekly *Culicoides* abundance, density and presence. Continue

Species	Year	*September* week=4; active=1.7-1.9; trap=10			*October* week=4-5; active=1.7-1.9; trap=10			*November* week=3-4; active=1.8-1.7; trap=10			*December* week=3-2; active=1.6-1.9; trap=10		
		**Density**	**Abund**	**Presence**	**Density**	**Abund**	**Presence**	**Density**	**Abund**	**Presence**	**Density**	**Abund**	**Presence**
C. stellifer$^{\beta'}$	I	202.53(101.18)	8101	100(4)	55.15(21.92)	2,206	100(4)	3.93(1.53)	118	67(2)	0(0)	0	0(0)
	II	56.45(14.87)	2258	100(4)	16.88(4.08)	844	100(5)	0.95(0.39)	38	75(3)	0.6(0.2)	12	100(2)
C. torreyae$^{\beta'}$	I	0(0)	0	0(0)	0(0)	0	0(0)	0(0)	0	0(0)	0(0)	0	0(0)
	II	0.03(0.03)	1	25(1)	0(0)	0	0(0)	0(0)	0	0(0)	0(0)	0	0(0)
C. variipennis$^{\beta}$	I	0(0)	0	0(0)	0(0)	0	0(0)	0(0)	0	0(0)	0(0)	0	0(0)
	II	0(0)	0	0(0)	0(0)	0	0(0)	0(0)	0	0(0)	0(0)	0	0(0)
C. venustus$^{\beta'}$	I	1.65(0.4)	66	100(4)	4.18(1.14)	167	100(4)	2(0.84)	60	100(3)	0.13(0.06)	4	100(3)
	II	2.28(0.53)	91	100(4)	3.36(0.77)	168	100(5)	1.03(0.27)	41	100(4)	0.75(0.29)	15	100(2)
C. villosipennis$^{\beta'}$	I	0(0)	0	0(0)	0.03(0.03)	1	25(1)	0(0)	0	0(0)	0(0)	0	0(0)
	II	0(0)	0	0(0)	0(0)	0	0(0)	0(0)	0	0(0)	0(0)	0	0(0)
C. alachua/ sanguisuga	I	0(0)	0	0(0)	0(0)	0	0(0)	0(0)	0	0(0)	0(0)	0	0(0)
	II	0(0)	0	0(0)	0(0)	0	0(0)	0(0)	0	0(0)	0.1(0.1)	2	50(1)
C. baueri	I	0(0)	0	0(0)	0(0)	0	0(0)	0(0)	0	0(0)	0(0)	0	0(0)
	II	0(0)	0	0(0)	0(0)	0	0(0)	0(0)	0	0(0)	0(0)	0	0(0)
C. beckae	I	0(0)	0	0(0)	0(0)	0	0(0)	0(0)	0	0(0)	0(0)	0	0(0)
	II	0(0)	0	0(0)	0(0)	0	0(0)	0(0)	0	0(0)	0(0)	0	0(0)
C. furens	I	0(0)	0	0(0)	0(0)	0	0(0)	0(0)	0	0(0)	0(0)	0	0(0)
	II	0(0)	0	0(0)	0(0)	0	0(0)	0(0)	0	0(0)	0(0)	0	0(0)
C. guttipennis	I	0(0)	0	0(0)	0(0)	0	0(0)	0(0)	0	0(0)	0(0)	0	0(0)
	II	0(0)	0	0(0)	0(0)	0	0(0)	0(0)	0	0(0)	0(0)	0	0(0)
C. nanus	I	0.03(0.03)	1	25(1)	0(0)	0	0(0)	0(0)	0	0(0)	0(0)	0	0(0)
	II	0(0)	0	0(0)	0(0)	0	0(0)	0(0)	0	0(0)	0(0)	0	0(0)
C. ousairani	I	0(0)	0	0(0)	0(0)	0	0(0)	0(0)	0	0(0)	0(0)	0	0(0)
	II	0(0)	0	0(0)	0(0)	0	0(0)	0(0)	0	0(0)	0(0)	0	0(0)
C. spp.	I	0(0)	0	0(0)	0(0)	0	0(0)	0(0)	0	0(0)	0(0)	0	0(0)
	II	0(0)	0	0(0)	0.02(0.02)	1	20(1)	0(0)	0	0(0)	0(0)	0	0(0)
**Total: Year-Month**	I	880.3(700.15)	8,803	-	291.6(98.24)	2,916	-	32.1(14.38)	321	-	0.7(0.4)	7	-
	II	260.5(94.05)	2,605	-	154.4(68.64)	1,544	-	21.5(6.56)	215	-	4.5(0.9)	45	-
**Total: Month**		142.6(52.55)	11,408	-	49.56(11.9)	4,460	-	7.66(2.42)	536	-	1.04(0.26)	52	-

Table S1.5. Monthly, year and total weekly *Culicoides* abundance, density and presence. Continue

Species	Year	*Species-Period*			*Species*		
		**Abund**	**Rel.abund**	**Presence**	**Abund**	**Rel.abund**	**Presence**
C. arboricola$^{\beta'}$	I	103	0.34	39(19)	131	0.25	33(32)
	II	28	0.13	27(13)			
C. bickleyi$^{\beta'}$	I	36	0.12	22(11)	154	0.3	25(24)
	II	118	0.54	27(13)			
C. biguttatus$^{\beta'}$	I	1,627	5.34	20(10)	3,360	6.44	23(22)
	II	1,733	7.99	25(12)			
C. chewaclae$^{\beta'}$	I	0	0	0(0)	18	0.03	5(5)
	II	18	0.08	10(5)			
C. crepuscularis$^{\beta'}$	I	10	0.03	12(6)	22	0.04	16(16)
	II	12	0.06	21(10)			
C. debilipalpis$^{\beta'}$	I	304	1	47(23)	344	0.66	40(39)
	II	40	0.18	33(16)			
C. haematopotus$^{\beta'}$	I	3,991	13.1	71(35)	7,042	13.5	79(77)
	II	3,051	14.07	88(42)			
C. hinmani$^{\beta'}$	I	15	0.05	12(6)	19	0.04	10(10)
	II	4	0.02	8(4)			
C. insignis$^{\beta'}$	I	27	0.09	6(3)	69	0.13	13(13)
	II	42	0.19	21(10)			
C. kirbyi$^{\beta'}$	I	8	0.03	6(3)	41	0.08	7(7)
	II	33	0.15	8(4)			
C. loisae$^{\beta'}$	I	5	0.02	10(5)	14	0.03	11(11)
	II	9	0.04	13(6)			
C. pallidicornis$^{\beta'}$	I	1,348	4.42	31(15)	5,931	11.37	34(33)
	II	4,583	21.13	38(18)			
C. paraensis$^{\beta'}$	I	34	0.11	24(12)	39	0.07	16(16)
	II	5	0.02	8(4)			
C. piliferus$^{\beta'}$	I	17	0.06	12(6)	135	0.26	14(14)
	II	118	0.54	17(8)			
C. pusillus	I	0	0	0(0)	1	<0.01	1(1)
	II	1	<0.01	2(1)			
C. scanloni$^{\beta'}$	I	18	0.06	8(4)	68	0.13	11(11)
	II	50	0.23	15(7)			
C. spinosus$^{\beta'}$	I	114	0.37	27(13)	424	0.81	30(29)
	II	310	1.43	33(16)			

Table S1.5. Monthly, year and total weekly *Culicoides* abundance, density and presence. Continue

Species	Year	*Species-Period*			*Species*		
		**Abund**	**Rel.abund**	**Presence %**	**Abund**	**Rel.abund %**	**Presence %**
C. stellifer[β']	I	21,838	71.66	73(36)	32,073	61.49	77(75)
	II	10,235	47.19	81(39)			
C. torreyae[β']	I	0	0	0(0)	10	0.02	5(5)
	II	10	0.05	10(5)			
C. variipennis[β]	I	8	0.03	4(2)	8	0.02	2(2)
	II	0	0	0(0)			
C. venustus[β']	I	944	3.1	86(42)	2,205	4.23	91(88)
	II	1,261	5.81	96(46)			
C. villosipennis[β']	I	11	0.04	10(5)	14	0.03	7(7)
	II	3	0.01	4(2)			
C. alachua/ sanguisuga	I	2	0.01	2(1)	7	0.01	5(5)
	II	5	0.02	8(4)			
C. baueri	I	3	0.01	6(3)	9	0.02	6(6)
	II	6	0.03	6(3)			
C. beckae	I	3	0.01	6(3)	3	0.01	3(3)
	II	0	0	0(0)			
C. furens	I	1	<0.01	2(1)	7	0.01	4(4)
	II	6	0.03	6(3)			
C. guttipennis	I	1	<0.01	2(1)	5	0.01	4(4)
	II	4	0.02	6(3)			
C. nanus	I	4	0.01	6(3)	5	0.01	4(4)
	II	1	<0.01	2(1)			
C. ousairani	I	2	0.01	4(2)	3	0.01	3(3)
	II	1	<0.01	2(1)			
C. spp.	I	1	<0.01	2(1)	2	<0.01	2(2)
	II	1	<0.01	2(1)			
**Total: Year-Month**	I	**30,475**	-	-	-	**58.42**	-
	II	**21,688**	-	-	-	**41.58**	-
**Total: Month**		-	-	-	**52,163**	**100**	-

References

1. Borkent, A. World species of biting midges (Diptera: Ceratopogonidae). 2016. *Last update May* **2016**, *16*, 2016.
2. Blanton, F.S.; Wirth, W.W. The sand flies (Culicoides) of Florida (Diptera: Ceratopogonidae). *Arthropods of Florida and Neighboring Land Areas* 1979, *10*, 1–204.
3. Nelder, M.P.; Swanson, D.A.; Adler, P.H.; Grogan, W.L. Biting Midges of the Genus Culicoides in South Carolina Zoos . *Journal of Insect Science* **2010**, *10*, 1–9, doi:10.1673/031.010.5501.
4. Kahle, D.; Wickham, H. ggmap: Spatial Visualization with ggplot2. *The R journal* **2013**, *5*, 144–161.
