# Supplementary material for: Tracking Community Timing: Pattern and Determinants of Seasonality in Culicoides (Diptera: Ceratopogonidae) in Northern Florida
Source: Viruses. 2020 Aug 25;12(9):931. doi: 10.3390/v12090931 (PMC7552033; doi:10.3390/v12090931)
Supplement: Supplementary file 1 [file viruses-12-00931-s001.zip › viruses-863910-for conversion-suppl/Supplementary 2_viruses-863910[July24].pdf]

Supplementary File 2: Culicoides β-diversity spatiotemporal dynamics

In: Tracking community timing: pattern and determinants of seasonality in *Culicoides* (Diptera: Ceratopogonidae) in northern Florida.

Agustín I. Quaglia [1,*], Erik M. Blosser [2], Bethany L. McGregor [1,2], Alfred E. Runkel IV [1], Kristin E. Sloyer [1], Dinesh Erram [1], Samantha M. Wisely [3] and Nathan D. Burkett-Cadena[1]

1 Florida Medical Entomology Laboratory, University of Florida, 200 9th St. SE, Vero Beach, FL 32962, USA;
2 United States Department of Agriculture, 1515 College Ave., Manhattan, Kansas, 66502, USA;
3 Department of Wildlife Ecology and Conservation, University of Florida, 110 Newins-Ziegler Hall, Gainesville, Florida, 32611, USA
* Correspondence: aquaglia@ufl.edu; Tel.: +01-772-226-6644 (AIQ)

Culicoides β-diversity spatiotemporal dynamics

The spatial contribution on the temporal organization of Culicoides community at local level was inspected using the three steps frame proposed by Angelar et al. [1] with certain modifications: 1) temporal structures in the design were extracted using Asymmetric eigenvector maps (*AEM*) instead of Principal Coordinate of Neighbor Matrices (*PCNM*), 2) these structures were derived into the species predictor matrices to account for the temporal scales in community change, 3) spatial structures were derived from the traps grid by Distance-based Moran's eigenvector maps (*dbMEM*) instead of *PCNM*, and 4) each temporal assemblage matrix was subjected to variance partitioning by spatial and environmental submodels.

Step 1: β-diversity temporal scales

Multivariate time series modelling was utilized to explore the time scales structure regarding change in *Culicoides* community composition among traps. Week-trap by species abundance matrices were transformed to Hellinger distance matrices. This transformation means that species abundance is divided by the total abundance of the vector (row) and then square transformed or applying the Chord transformation to the square root abundance data. Subsequently, Euclidean distances between vectors are calculated. Hellinger distance transformation fulfilled the necessary properties to *β*-diversity assessment [2].

Time sampling schedule (101 weeks) represented a linear and directional trend that can be subject to a fractal decomposition (i.e., Fourier transformation). Distance based Moran's eigenvectors maps (*dbMEM*) have been widely used [1,3], however, directional spatial (stream flow) or temporal processes (phenology or any temporal correlation structures) are robustly captured by *AEM* because monotonic trends (sampling trend), prolonged, intermediate and brief oscillations are accounted in the response variables. Linear trends cannot be depicted with *MEM* (Moran's eigenvectors maps) since detrended response is required [3,4]. *AEM* turns a series of sine-wave time derived (i.e., feasible plethora of time scales structures) into explanatory variables for modelling oscillations in the species composition matrix through *RDA* [5]. In this way, influences of a sample t_0 on future samples $t_{1\ldots}t_n$ are accounted, but not in the opposite way (t_1-t_0), then control on the correlation structure is consistently accounting by the directionality inherent to the event. Total sets of t_{n-1} *AEM* eigenfunctions (*AEMs*) variables partition the time frame. Importantly, inferences from scales are independent as *AEMs* are orthogonal dimensions.

In addition, *AEMs* are sorted in positive and negative correlations structures. Positive *AEMs* represent temporal autocorrelation for the environmental-time related fluctuations in *β*-diversity, whereas negative *AEMs* could inform about short scale *β*-diversity variations driven by biotic interaction process (competition and predation)[6].

To accomplish *AEMs* estimation, easiness of exchange between successive dates is mandatory by computing weights in the time matrix edges (1 for dependent dates and 0 when there is not influence; [7](Figure 4)). Edges values were set using the non-significant shortest time span among sampling dates (autocorrelation) as threshold with multivariate Mantel correlogram (*mMC*). The longest threshold was selected among the traps as the time scales might be comparable between traps. Mantel correlogram was run in *vegan* packages [8] with permutations (*n*= 999) and *AEMs* with Moran's values in *adespatial* packages [9].

Model selection by forward selection [10] was done to retain temporal structures behind the change in species composition, where separate redundancy analyses (*RDA*) were run for positive and negative *AEM* subsets. Adjusted determination coefficient (R^2_{adj}) was calculated with 9999 permutations and selection stopped after reaching previous R^2_{adj} value or value $p \geq 0.05$ (*adespatial* packages). Subsequently, *RDA* were run again with the selected AEMs and after 9999 permutation linear combination scores (*Lc*) of significant axes (RDA_{axis}) were retained.

Step 2: Species predictor matrices

Species predictor matrices accounting for temporal scales in community change Spearman rank correlation (*Rho*) between the abundance of each species and *Lc* was used to assess how species were related to temporal scales in each trap. Relevant species were those showing significant (α=0.05) |*Rho*| and represent the species temporal-trend matrices [1].

Step 3: Spatial scales involved in the temporal β-diversity pattern

Phenology patterns in *Culicoides* composition derived for the trap spatial distributions was explored by *dbMEM*. This spatial analytical tool was selected because it draws linearly independent descriptors of spatial scales (alike *AEM*), depicts a wide range of spatial scales and is suitable to model regular and irregular sampling designs [5]. Traps location were irregular (main text: *Materials and methods*; Figure S1.1). Also, *dbMEM* has showed proper detection of spatial autocorrelation since the eigenvalues of connected sites (after truncation) will be greater than Morans's I expectations. The maximum distance of the minimum spanning tree was used to estimate the truncation distance holding all the traps connected. Through this threshold all possible *dbMEM* eigenfunctions (*dbMEMs*) were extracted with geographical coordinates of traps. Edge threshold and *dbMEMs* were computed with *adespatial* packages.

Temporal trend matrices served as response layout and *dbMEMs* were the explanatory spatial scales in *RDA*. Although time average species abundance by trap could be used as response layer to describe spatial structures in the community organization, species temporal-trend matrices (*Step 2*) derived from correlation scores have proven to represent better species temporal change in metacommunity analyses [1]. Spatial trend (geographical coordinates) in response data was checked by permutation and detrended and the successive steps follow selection of scales described in *Step 1*.

Step 4: Variance partitioning by spatial and environmental submodels

Variation partitioning analysis was described in section *2.4. Culicoides β-diversity temporal profile* in the main manuscript.

Results and discussion

Tracking the time dependency in *Culicoides* community composition showed a similar correlation time span across traps. β-diversity measures within a 72 days frame were significantly autocorrelated (Table S2.1; $\bar{x}$=67.55 days, *sd*=4.93, range= [57.61; 71.9]) and inference at shorter time frame could not be supported. Figure S2.2 shows *mMC*'s plots in two traps as an example.

Unique *AEMs* (t_{n-1}=100) time set were built using the longest time frame setting (72 days) in the autocorrelation analysis as threshold. After forward-back selection only positive *AEMs* were selected. Three canonical axes were enough to explain 30-60 % variability for the changing *Culicoides* community composition (Table S2.2). These axes informed three independent temporal patterns for all traps but trap 12 had only two and trap 16 had four (Table S2.2).

Table S2.3 summarizes temporal scales of each canonical axis. Twelve species participated in the composition change at annual scale, but four species were of the highest magnitude in all traps (*C. stellifer, C. debilipalpils, C. haematopotus* and *C. pallidicornis*; Figure S2.2). In the biannual scale 9 species were involved for a subset of traps (Figure S2.3). Quarterly variation in β-diversity (16 *species*) variation was mainly related to *C. venustus* (Figure S2.4). *Culicoides* composition variation at bimonthly scale was significant in the trap 16 (Table S2.2), however the autocorrelation truncation threshold for this trap (63 days) turned suspicious its significance.

Spatial scales or spatial autocorrelation were not apparent on the β-diversity time profiles above described. Then, temporal dynamics in *Culicoides* β-diversity is spatially homogeneous. First, distance between traps depicted a similar distribution (Figure S2.5) and the minimum distance that keeps all traps connected was 606m (i.e.: truncation distance; Figure S2.5, red horizontal line). Second, no significant spatial trend was proven in the three species temporal-trend matrices against geographical coordinates by permutation ($p \geq 0.05$). Importantly, none of three *dbMEMs* coming from the traps spatial configuration explained the response matrices ($p \geq 0.05$) with the three spatial procedure based on Hassle-Free *dbMEM* (*quickMEM* function), *dbMEM* coding step by step and *PCNM* (*quickPCNM* function) proposed in Borcad et al 2018. Also, *mMC* failed to reveal significant spatial autocorrelation after the first-class index (328.7m; Figure S2.6). This suggests that species do not switch recruitment sites among time, and that sites connectivity is high. Host space use and midge dispersion may blur the expected spatial structures related to larval habitats preferences, temporal variation in habitat availability or temporal switch in habitat use.

Given the spatial extent accounted for in the sampling area (180.53ha) and the mobile nature of *Culicoides* adults, it could be less likely to find strong enough spatial component in the temporal dynamic of *Culicoides* composition at local scale. Moreover, local dispersion in adults can turn less evident the spatial relationship between larval habitat partitioning among species [11,12] and the foraging behavior of adult *Culicoides* [13]. The distance *Culicoides* can disperse without wind assistance (~2-6 km [13,14]) was between the minimal distance that keep connected all trapping sites (Figure S2.5), then temporal variability in larval habitat availability or the switch in habitat use were not detected.

Alternately, the limited host dispersion may explain the absence of spatiotemporal structures as host availability also drives the distribution of mosquitoes and *Culicoides* [14–20]. In this highly fenced preserve, native and exotic ruminants are the main blood source for *Culicoides* [21].Fences plus food supplementation in stations could increase host availability by modifying host range use [22] and midges exploit them uniformly in time. Furthermore, it is recognized how the heterogeneous distribution of pathogen, vectors and hosts among time and space in the environment leads to transmission hotspot [23–26]. Locally, it seems that the spatial distribution of *BTV-EHDV* transmission hotspots do not change in time as *Culicoides* β-diversity was spatially homogeneous. However, the dynamic of hotspot may be relevant within seasons as

spatial clusters of *Culicoides* are highly variable between nights [18]. *Consequently, Culicoides β-diversity temporal profile* was analyzed with averaged week species composition data.

Table S2.1. Minimum number of days without time autocorrelation by multivariate correlogram.

Trap id	*Distance class*	*Class index (days)*	*Mantel correlation*	*p Corrected*
**1**	10	71.91	0.02	0.108
**2**	10	71.19	0.02	0.09
**5**	10	71.91	0.01	0.172
**10**	10	71.91	0.02	0.096
**11**	10	71.91	0.01	0.116
**12**	9	64.76	0.01	0.093
**15**	9	64.76	0.02	0.074
**16**	9	64.76	0.02	0.062
**17**	9	64.76	0.02	0.058
**18**	9	57.61	0.02	0.106

p corrected: adjusted *p* after multiple testing with Holm's method.

Table S2.2. Number of temporal scales and *Culicoides* composition change explained variation

Trap	*Number of* RDA_{axis}	p	R^2	R^2_{adj}
**1**	3	0.001	0.64	0.59
**2**	3	0.001	0.55	0.47
**5**	3	0.001	0.57	0.50
**10**	3	0.001	0.42	0.36
**11**	3	0.001	0.57	0.48
**12**	2	0.001	0.37	0.30
**15**	3	0.001	0.52	0.45
**16**	4	0.001	0.55	0.46
**17**	3	0.001	0.61	0.54
**18**	3	0.001	0.56	0.51

p: *p-value* after permutation analysis (n=1,000). R^2_{adj}: Adjusted determination coefficient. RDA_{axis}: significant axes in redundancy analysis after regressing with the *AEMs* subset (permutation analysis, n=1,000).

Table S2.3. Depicting temporal scales in *Culicoides* β-diversity

Temporal Scale	RDA_{axis}	*Number of Oscillations*	*Weeks* $\bar{x}$ *(sd)*	*Weeks range*
**Annual**	RDA_1	2	52 (2.83)	50-54
**Biannual**	RDA_2	4	26 (8.98)	17-36
**Quarterly**	RDA_3	6	17.33 (3.33)	13-22
**Bimonthly**	RDA_4	11	9.45 (4.41)	5-17

RDA_{axis}: coded as Figure S2.2 - Figure S2.4. *Week range*: is the minimum and maximum oscillation durations (weeks) in the linear combinators scores of temporal fluctuations for a given RDA_{axis}

Figure S2.1. *Culicoides* composition and time autocorrelation: multivariate Mantel correlogram

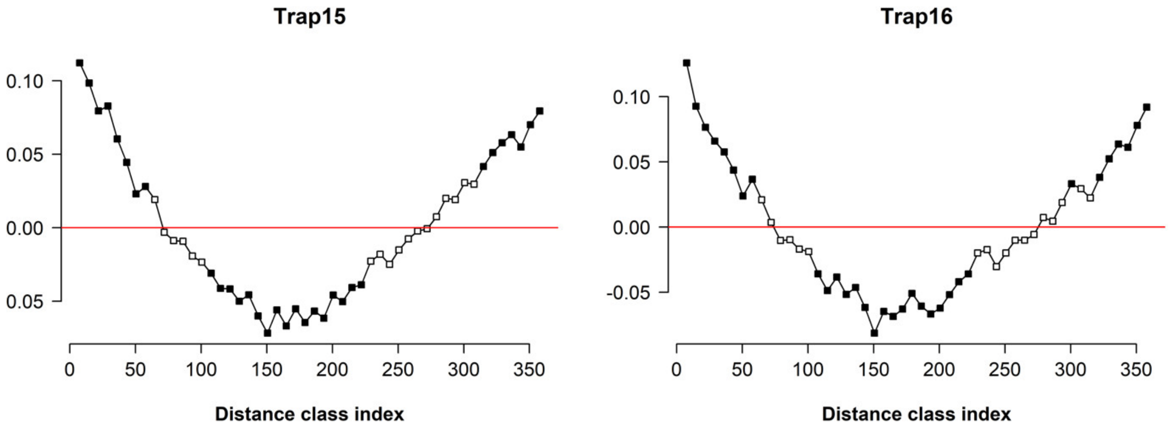

Distance class index: distance (days) between sampling dates. Above red line it is showed positive Mantel correlation values, indeed postive autocorrelation. Black filled squares identify significant Mantel correlation values ($p \leq 0.05$).

Figure S2.2. *Culicoides* compositional change at Annual scale: Linear combinations scores series (above) and group of species involved (below).

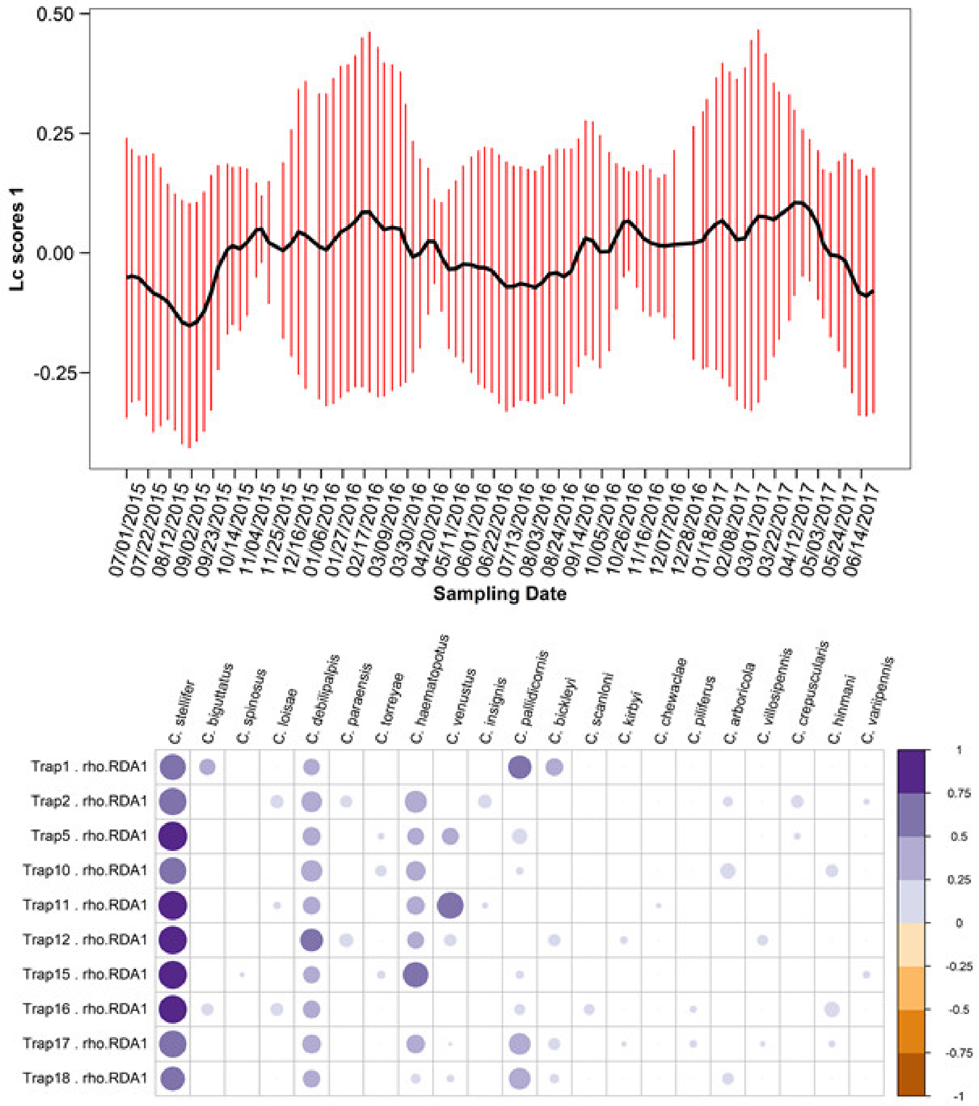

Top plot: Black line: mean *Lc* value; red vertical bars is standart deviations from mean *Lc* score among traps. *Lc* scores: Linear combination scores are the eigenvalues for a given RDA_{axis}. Bottom plot: Culicoides Spearman Rank correlation (*Rho*) between traps at the corresponding temporal scale. Only significant (p<0.05) correlation are represented. Size and transparency show magnitude in |*Rho*|.

Figure S2.3. *Culicoides* compositional change at Biannual scale: Linear combinations scores series (above) and group of species involved (below).

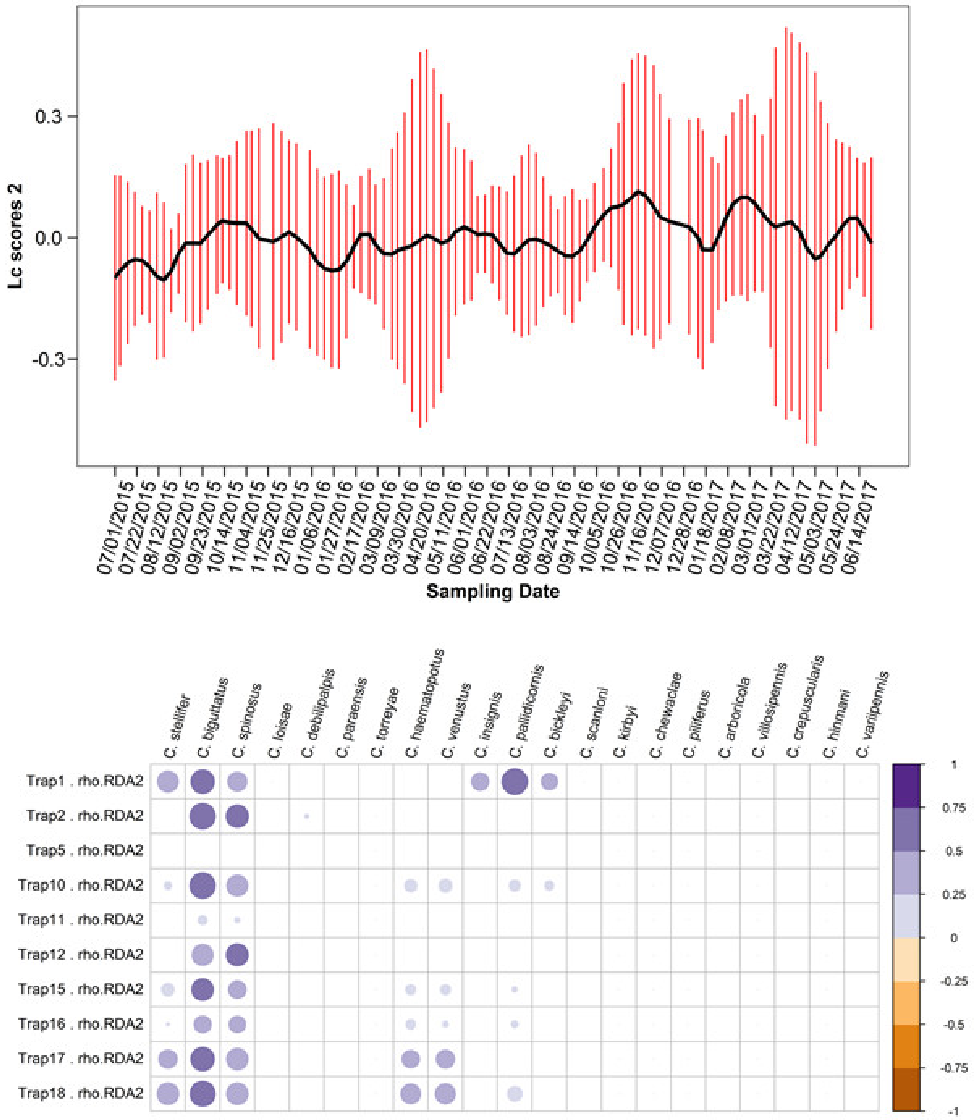

Details in Figure S2.2

Figure S2.4. *Culicoides* compositional change at Quarterly scale: Linear combinations scores series (above) and group of species involved (below).

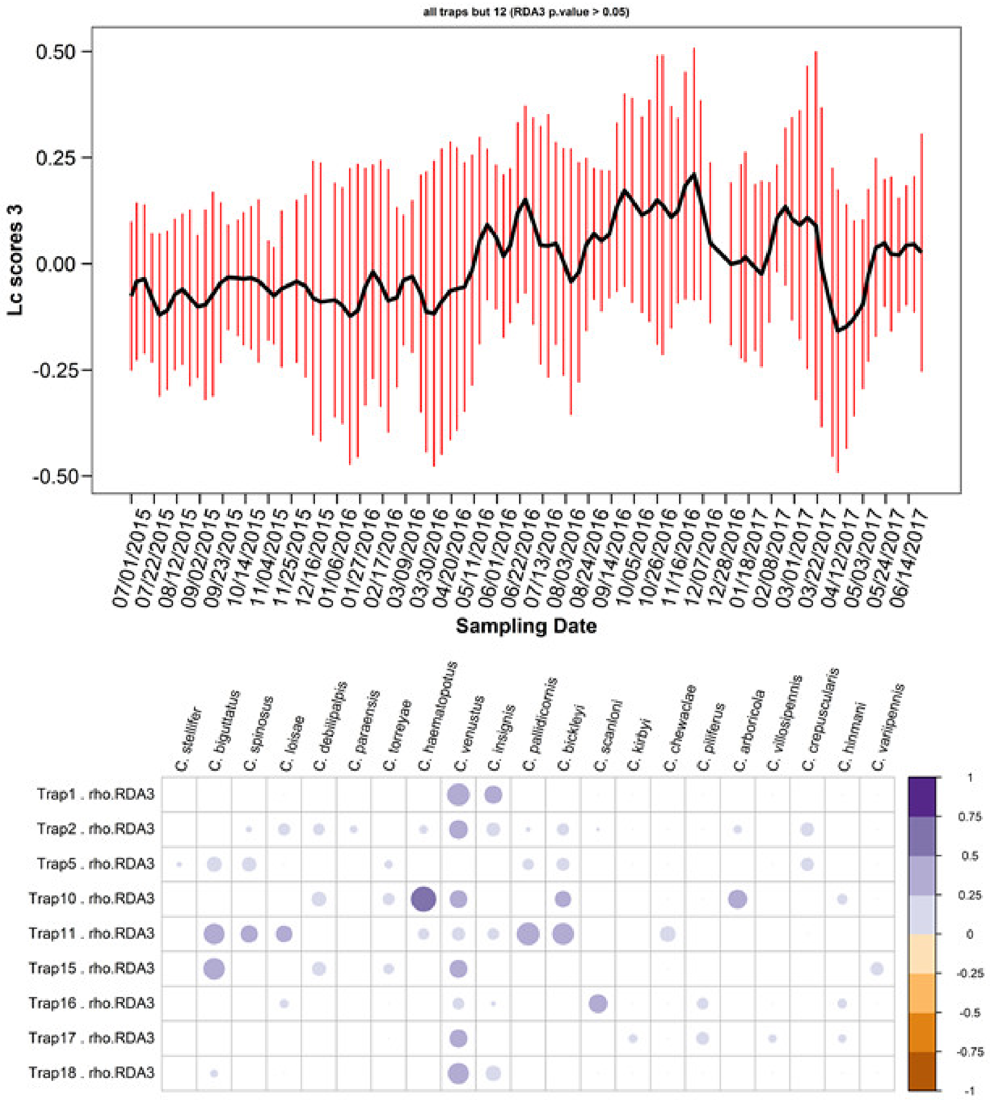

Details in Figure S2.2

Figure S2.5. Traps spatial distribution: distance between traps.

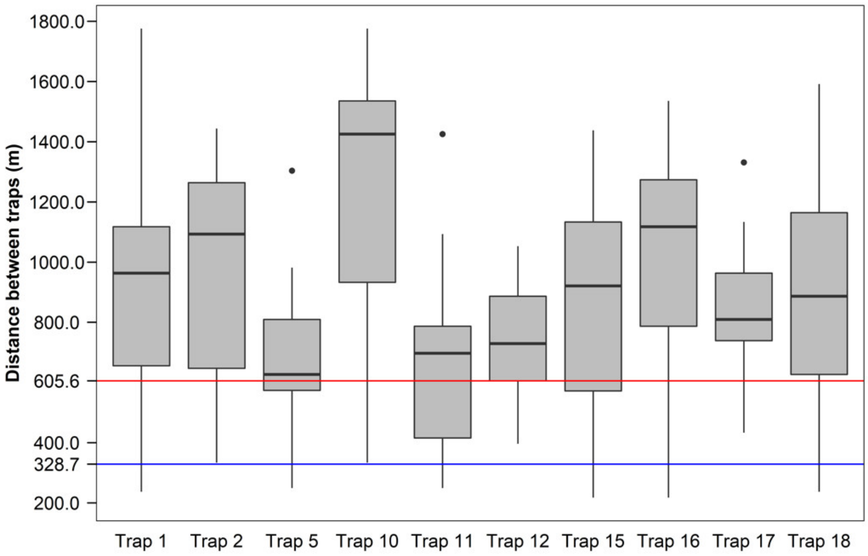

Red line: truncation distance holding all traps connected by the maximum distance of the minimum spanning tree (605.6m). Blue line: minimun distance class index in multivariate Mantel correlogram (Figure S2.6)

Figure S2.6. Spatial autocorrelation in temporal scales for *Culicoides* β-diversity: multivariate Mantel correlogram.

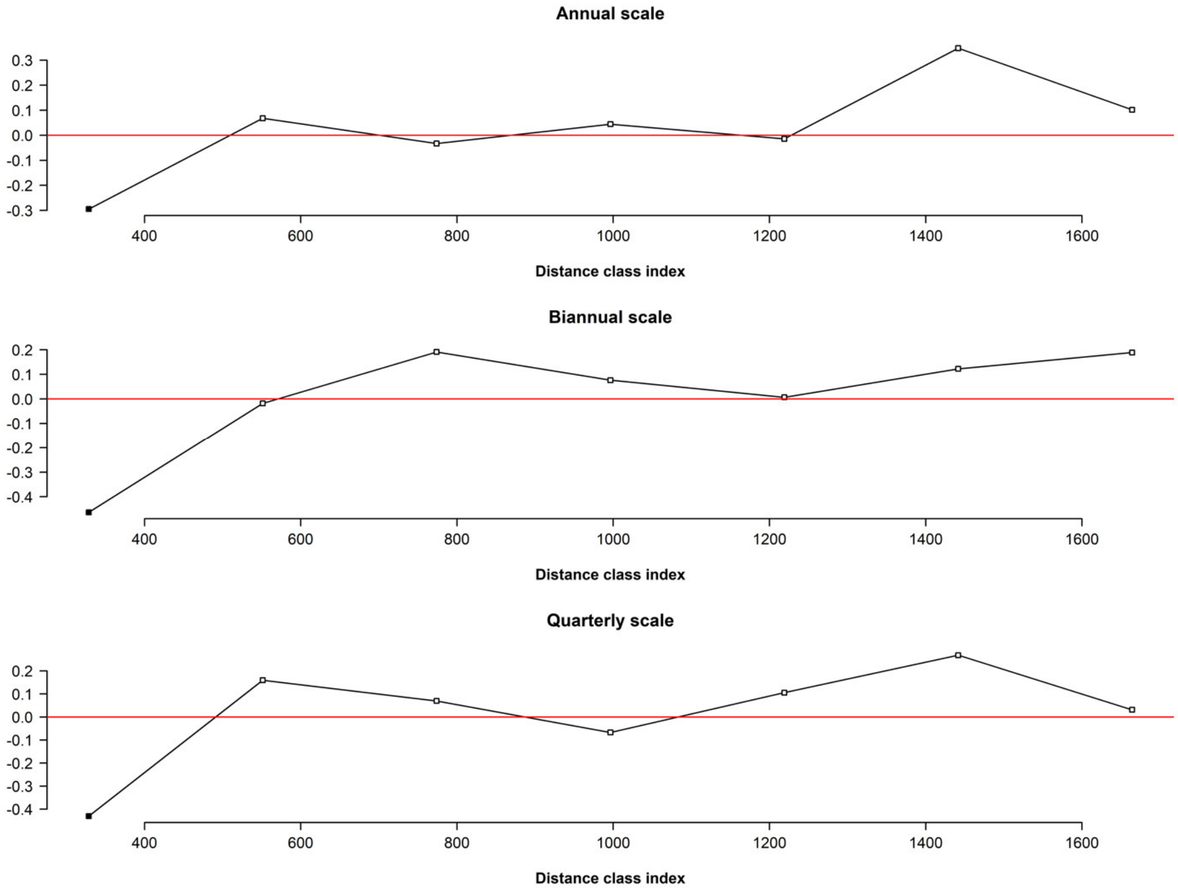

Distance class index: distance (m) between traps. Details in Figure S2.1.

References

1. Angeler, D.G.; Göthe, E.; Johnson, R.K. Hierarchical Dynamics of Ecological Communities: Do Scales of Space and Time Match? *PLoS ONE* **2013**, *8*, doi:10.1371/journal.pone.0069174.
2. Legendre, P.; de Cáceres, M. Beta diversity as the variance of community data: Dissimilarity coefficients and partitioning. *Ecology Letters* **2013**, *16*, 951–963, doi:10.1111/ele.12141.
3. Baho, D.L.; Futter, M.N.; Johnson, R.K.; Angeler, D.G. Assessing temporal scales and patterns in time series: Comparing methods based on redundancy analysis. *Ecological Complexity* **2015**, *22*, 162–168, doi:10.1016/j.ecocom.2015.04.001.
4. Blanchet, F.G.; Legendre, P.; Borcard, D. Modelling directional spatial processes in ecological data. *Ecological Modelling* **2008**, *215*, 325–336, doi:10.1016/j.ecolmodel.2008.04.001.
5. Borcard, D.; Gillet, F.; Legendre, P. Spatial analysis of ecological data. In *Numerical ecology with R*; Springer, 2018; pp. 299–367.
6. Blanchet, F.G.; Roslin, T.; Kimura, M.T.; Huotari, T.; Kaartinen, R.; Gripenberg, S.; Tack, A.J.M. Related herbivore species show similar temporal dynamics. *Journal of Animal Ecology* **2018**, *87*, 801–812, doi:10.1111/1365-2656.12807.
7. Legendre, P.; Gauthier, O. Statistical methods for temporal and space-time analysis of community composition data. *Proceedings of the Royal Society B: Biological Sciences* **2014**, *281*, doi:10.1098/rspb.2013.2728.
8. Oksanen, J.; Blanchet, F.G.; Kindt, R.; Legendre, P.; O'hara, R.B.; Simpson, G.L.; Solymos, P.; Stevens, M.H.H.; Wagner, H. Vegan: community ecology package. R package version 1.17-4. *URL http://CRAN. R-project. org/package= vegan* **2010**.
9. Dray, S.; Blanchet, G.; Borcard, D.; Clappe, S.; Guenard, G.; Jombart, T. adespatial: multivariate multiscale spatial analysis.–R package ver. 0.1-1 2018.
10. Blanchet, F.G.; Legendre, P.; Borcard, D. Forward selection of explanatory variables. *Ecology* **2008**, *89*, 2623–2632, doi:10.1890/07-0986.1.
11. Blackwell, A.; Lock, K.A.; Marshall, B.; Boag, B.; Gordon, S.C. The spatial distribution of larvae of Culicoides impunctatus biting midges. *Medical and Veterinary Entomology* **1999**, *13*, 362–371, doi:10.1046/j.1365-2915.1999.00180.x.
12. Erram, D.; Blosser, E.M.; Burkett-Cadena, N. Habitat associations of Culicoides species (Diptera: Ceratopogonidae) abundant on a commercial cervid farm in Florida, USA. *Parasites and Vectors* **2019**, *12*, 1–13, doi:10.1186/s13071-019-3626-1.
13. Purse, B.V.; Carpenter, S.; Venter, G.J.; Bellis, G.; Mullens, B.A. Bionomics of Temperate and Tropical Culicoides Midges: Knowledge Gaps and Consequences for Transmission of Culicoides -Borne Viruses . *Annual Review of Entomology* **2015**, *60*, 373–392, doi:10.1146/annurev-ento-010814-020614.
14. Kluiters, G.; Swales, H.; Baylis, M. Local dispersal of palaearctic Culicoides biting midges estimated by mark-release-recapture. *Parasites and Vectors* **2015**, *8*, doi:10.1186/s13071-015-0658-z.
15. Sanders, C.J.; Shortall, C.R.; Gubbins, S.; Burgin, L.; Gloster, J.; Harrington, R.; Reynolds, D.R.; Mellor, P.S.; Carpenter, S. Influence of season and meteorological parameters on

flight activity of Culicoides biting midges. *Journal of Applied Ecology* **2011**, *48*, 1355–1364, doi:10.1111/j.1365-2664.2011.02051.x.
16. Purse, B. v.; Falconer, D.; Sullivan, M.J.; Carpenter, S.; Mellor, P.S.; Piertney, S.B.; Mordue Luntz, A.J.; Albon, S.; Gunn, G.J.; Blackwell, A. Impacts of climate, host and landscape factors on Culicoides species in Scotland. *Medical and Veterinary Entomology* **2012**, *26*, 168–177, doi:10.1111/j.1365-2915.2011.00991.x.
17. Burkett-Cadena, N.D.; McClure, C.J.W.; Estep, L.K.; Eubanks, M.D. What drives the spatial distribution of mosquitoes? *Ecosphere* **2013**, *4*, doi:10.1890/ES13-00009.1.
18. Kirkeby, C.; Bødker, R.; Stockmarr, A.; Lind, P. Spatial abundance and clustering of Culicoides (Diptera: Ceratopogonidae) on a local scale. *Parasites and Vectors* **2013**, *6*, doi:10.1186/1756-3305-6-43.
19. Diarra, M.; Fall, M.; Lancelot, R.; Diop, A.; Fall, A.G.; Dicko, A.; Seck, M.T.; Garros, C.; Allène, X.; Rakotoarivony, I.; et al. Modelling the abundances of two major culicoides (Diptera: Ceratopogonidae) species in the niayes area of senegal. *PLoS ONE* **2015**, *10*, doi:10.1371/journal.pone.0131021.
20. Grimaud, Y.; Guis, H.; Chiroleu, F.; Boucher, F.; Tran, A.; Rakotoarivony, I.; Duhayon, M.; Cêtre-Sossah, C.; Esnault, O.; Cardinale, E.; et al. Modelling temporal dynamics of Culicoides Latreille (Diptera: Ceratopogonidae) populations on Reunion Island (Indian Ocean), vectors of viruses of veterinary importance. *Parasites and Vectors* **2019**, *12*, 1–17, doi:10.1186/s13071-019-3812-1.
21. McGregor, B.L.; Stenn, T.; Sayler, K.A.; Blosser, E.M.; Blackburn, J.K.; Wisely, S.M.; Burkett-Cadena, N.D. Host use patterns of Culicoides spp. biting midges at a big game preserve in Florida, U.S.A., and implications for the transmission of orbiviruses. *Medical and Veterinary Entomology* **2019**, *33*, 110–120, doi:10.1111/mve.12331.
22. Dinh, E.T.N.; Cauvin, A.; Orange, J.P.; Shuman, R.M.; Wisely, S.M.; Blackburn, J.K. Living la Vida T-LoCoH: Site fidelity of Florida ranched and wild white-tailed deer (Odocoileus virginianus) during the epizootic hemorrhagic disease virus (EHDV) transmission period. *Movement Ecology* **2020**, *8*, 1–9, doi:10.1186/s40462-020-00200-2.
23. Yaremych, S.A.; Novak, R.J.; Raim, A.J.; Mankin, P.C.; Warner, R.E. Home range and habitat use by American crows in relation to transmission of West Nile virus. *Wilson Bulletin* **2004**, *116*, 232–239, doi:10.1676/03-104.
24. Reisen, W.K. Landscape Epidemiology of Vector-Borne Diseases. *Annual Review of Entomology* **2010**, *55*, 461–483, doi:10.1146/annurev-ento-112408-085419.
25. Paull, S.H.; Song, S.; McClure, K.M.; Sackett, L.C.; Kilpatrick, A.M.; Johnson, P.T.J. From superspreaders to disease hotspots: Linking transmission across hosts and space. *Frontiers in Ecology and the Environment* 2012, *10*, 75–82.
26. Janousek, W.M.; Marra, P.P.; Kilpatrick, A.M. Avian roosting behavior influences vector-host interactions for West Nile virus hosts. *Parasites and Vectors* **2014**, *7*, doi:10.1186/1756-3305-7-399.
